# Instrument Calibration of the *Interface Region Imaging Spectrograph* (IRIS) Mission

**DOI:** 10.1007/s11207-018-1364-8

**Published:** 2018-10-30

**Authors:** J.-P. Wülser, S. Jaeggli, B. De Pontieu, T. Tarbell, P. Boerner, S. Freeland, W. Liu, R. Timmons, S. Brannon, C. Kankelborg, C. Madsen, S. McKillop, J. Prchlik, S. Saar, N. Schanche, P. Testa, P. Bryans, M. Wiesmann

**Affiliations:** 1 Lockheed Martin Solar & Astrophysics Laboratory, Lockheed Martin Advanced Technology Center, Org. A021S, Bldg. 252, 3251 Hanover St., Palo Alto, CA 94304 USA; 2 Department of Physics, Montana State University, Bozeman, P.O. Box 173840, Bozeman, MT 59717 USA; 3 Harvard-Smithsonian Astrophysical Observatory, 60 Garden Street, Cambridge, MA 02138 USA; 4 High Altitude Observatory, National Center for Atmospheric Research, P.O. Box 3000, Boulder, CO 80307 USA; 5 Institute of Theoretical Astrophysics, University of Oslo, P.O. Box 1029, Blindern, Oslo, Norway; 6 Bay Area Environmental Research Institute, NASA Research Park, Mailstop 18-4, Moffett Field, CA 94035-0001 USA; 7 Present Address: National Solar Observatory, Pukalani, HI USA; 8 Present Address: Apple Inc., Cupertino, CA USA; 9 Present Address: University of St Andrews, St Andrews, Scotland UK

**Keywords:** Instrumentation: calibration

## Abstract

The *Interface Region Imaging Spectrograph* (IRIS) is a NASA small explorer mission that provides high-resolution spectra and images of the Sun in the 133 – 141 nm and 278 – 283 nm wavelength bands. The IRIS data are archived in calibrated form and made available to the public within seven days of observing. The calibrations applied to the data include dark correction, scattered light and background correction, flat fielding, geometric distortion correction, and wavelength calibration. In addition, the IRIS team has calibrated the IRIS absolute throughput as a function of wavelength and has been tracking throughput changes over the course of the mission. As a resource for the IRIS data user, this article describes the details of these calibrations as they have evolved over the first few years of the mission. References to online documentation provide access to additional information and future updates.

## Introduction

The *Interface Region Imaging Spectrograph* (IRIS) is a NASA small explorer mission launched on 27 June 2013. Its primary objective is to understand how the solar atmosphere is energized. IRIS observes high-resolution spectra and images in the 133 – 141 nm and 278 – 283 nm wavelength bands focused on the chromosphere and transition region of the Sun. The IRIS instrument consists of a Cassegrain telescope that feeds a dual-range spectrograph and a slit-jaw imager. The telescope secondary mirror is actuated to compensate for spacecraft jitter and to scan the spectrograph slit across a field of view of up to 130 arcsec. The far-ultraviolet (FUV) and near-ultraviolet (NUV) spectrographs share a single, 175 arcsec tall slit. The FUV spectrograph has two detectors; the FUV-S and the FUV-L CCDs cover the 133.17 – 135.84 and 138.9 – 140.7 nm ranges, respectively. The NUV spectrograph has a single CCD that covers the 278.27 – 283.51 nm range. The slit-jaw imager is fed by light reflected off the spectrograph slit jaws. It has separate FUV and NUV branches that feed different halves of the same CCD detector. Both paths use the same filter wheel for passband selection. The NUV path includes a birefringent Šolc filter that reduces the bassband to about 0.4 nm. The IRIS mission and instrument are described in more detail in De Pontieu *et al.* (2014) and Wülser *et al.* (2012).

The goal of this article is to describe the various calibrations that are either routinely applied to the IRIS data or that are otherwise important to the users of IRIS science data. Some of the calibrations have evolved substantially since the mission article (De Pontieu *et al.*, 2014) was published. All calibrations have specific characteristics and limitations that may affect the proper interpretation of the IRIS data and therefore warrant a more detailed discussion. The article may also provide lessons learned for the calibration procedures of future missions.

Recent solar physics science missions have typically chosen one of two different approaches to the distribution of the science data and their calibration: (1) Distribute raw data (or nearly raw data) and provide the user with the software necessary to calibrate that raw data. (2) Distribute data that are already calibrated. Both approaches have their advantages and disadvantages. The former approach puts the burden on the data user, requiring possibly resource-intensive calibration steps to be performed by the end user. However, it allows the user to apply the most up-to-date calibrations to a given dataset. The second approach does not burden the user with the calibration and requires less insight into the calibration steps by the user. On the other hand, the calibration of some of the mission archive may be outdated at the time the data are being used. Substantial improvements in the calibration procedures require reprocessing of the whole mission archive, which can become a major burden to the mission team.

The IRIS project has chosen the second approach, partly because of the complexity and memory requirements of the calibrations necessary for the proper interpretation of the IRIS science data and partly to take advantage of the existing *Solar Dynamics Observatory* (SDO) / *Atmospheric Imaging Assembly* (AIA) data calibration pipeline. The primary science data product for the scientist is the calibrated Level 2 data. The IRIS Level 2 mission archive has been reprocessed several times since launch and in 2017 with the updated calibrations described in this article.

This article is organized into sections for each of the main calibration steps: dark calibration (Section 2), scattered light correction (Section 3), flat fielding (Section 4), geometric correction (Section 5), wavelength calibration (Section 6), and image alignment (Section 7). Most of the described calibrations are applied during the creation of the Level 2 data. The descriptions include a few calibration issues that have been identified but are not currently being corrected, for example, spectral burn-in in the vicinity of the C ii lines (Section 4.3). These sections are followed by a discussion of the IRIS absolute throughput calibration and the sensitivity change of IRIS with time (Section 8). The spectral response and absolute throughput calibrations are not applied to the Level 2 data, but there are software tools that provide the user with mission time dependent spectral response and absolute throughput calibrations. Later sections briefly discuss other miscellaneous calibrations that are of interest to the data user (Section 9), outline the Level 2 data processing pipeline (Section 10), and comment on remaining calibration issues and known data peculiarities (Section 11). The article concludes with a brief summary.

## Dark Calibration

IRIS dark frames (integrations with the shutter closed) show a residual signal coming from two sources: an electronic pedestal introduced as a base level and the dark current. Both of these contributions are sensitive to their thermal environment. The latter is also sensitive to integration time and summing level. IRIS has many observation modes and summing schemes, and so to avoid the need for extensive daily dark calibrations, a model dark frame is used. Prelaunch and subsequent (approximately monthly) dark observations of varying dark integration times and summing schemes were taken to measure the contributions to the dark level. A model for the shape and level of the dark frame has been developed from these observations over the course of the mission and continues to be modified and improved regularly. This dark model is then used instead of actual dark frames. The dark model and how it was developed and calibrated is described below.

### Observations, Processing, and Calibration

To gather the data needed for calibrating the dark model, regular (approximately monthly) dark images are taken in two observing sequences. One focuses on unsummed ($1\times 1$) darks, taken with four dark integration times, $t_{\mathrm{int}}$, from 0.09 s to 30 s, over at least one spacecraft orbit. The shortest integration images (≈ 0 s) are used to track the thermal dependence of the pedestal level over the orbit. They are also used to construct an averaged “basal” dark, free of particle hits and hot pixels. The basal dark essentially represents the pixel-to-pixel variation component of the dark correction. The second observing sequence concentrates on summed data, taking darks at three integration times over all summing modes. These data are useful to establish summing dependencies and also to establish how the shape of the dark current surface varies with summing and $t_{\mathrm{int}}$. In practice, the active area of the CCDs are extracted for each read port, the image median smoothed, and all pixels $>4\sigma $ above local background replaced by that background average. The average level is then taken as a fiducial to match.

Initial calibration of the dark frame levels was discussed in De Pontieu *et al.* (2014), where they gave the model for the total dark level, $D$, in read port $j$ as

1 $$ D_{j} = P_{j}\bigl[T_{\mathrm{CEB}j}(t-\delta t_{j})\bigr] + \mathrm{e}^{(a_{j} + b_{j} T_{\mathrm{CCD}j})} n_{x} n_{y} t_{\mathrm{int}} + \Delta D _{j}(x, n_{x}, n_{y}, t_{\mathrm{int}}). $$

Here, $P_{j}$ is the pedestal level in read port $j$, which is a function of the camera electronics box (CEB) temperature, $T_{ \mathrm{CEB}j}$, time lagged by $\delta t_{j}$. The second term gives the average dark-current rate, which is the product of an exponential dependence on the CCD temperature, $T_{\mathrm{CCD}j}$, the amount of on-chip summing, $n_{x} n_{y}$, and the time between CCD reads (*i.e.* $t_{\mathrm{int}}$). The final term, $\Delta D$, models the change in the shape of the dark in the wavelength (*i.e.* $x$) direction as $t_{\mathrm{int}}$ and summing are increased, from flat for $t_{\mathrm{int}} \approx 0$ s and 1×1 summing, to roughly bilinear in $x$, rising first quickly and then more gradually away from the read-out point. The full amplitude of the pattern is $\Delta D \approx 10$ data numbers (DN) for the FUV ports with $n_{x} n_{y} = 32$. In practice, $D_{j}$ is computed for each port and added to the appropriate port of a basal dark. This basal dark was constructed by averaging 30 $t_{\mathrm{int}} \approx 0$ s images, after the pedestal $P_{j}$, particle hits, and hot pixels were removed.

After more on-orbit data had been accumulated, it became clear that the model above was incomplete. There were small differences in the predicted and observed dark levels for certain summing schemes. The latter was found to be due to an additional noise source dependent on $n_{x}$ alone. There were also small differences, differing from port to port and varying quasi-periodically in time, when the average model dark level was compared with actual darks. A long-term trend function, $L_{j}$, was developed to attempt to model and predict these long-timescale variations.

The revised dark model, $D'_{j}$, can thus be written as

2 $$ D'_{j} = D_{j} + c_{j}(n_{x}) + L_{j}(t), $$

where $c_{j} = 0$ for $n_{x}=1$.

**Figure 1:**
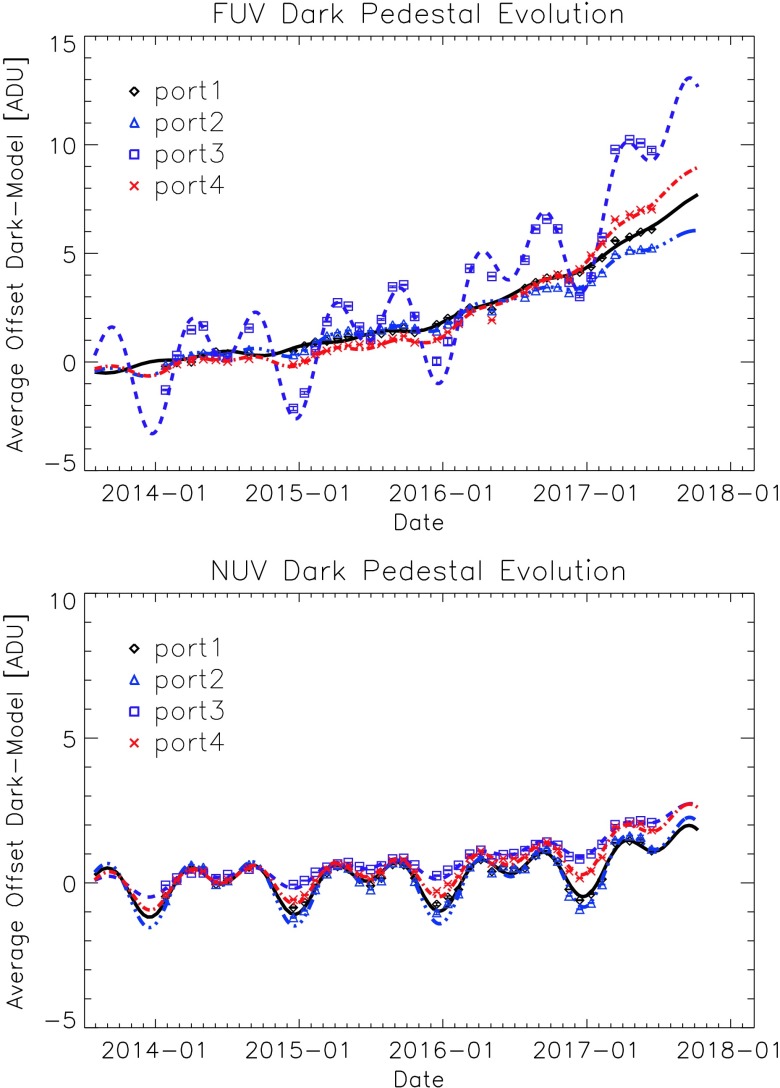
Average data number (DN or ADU) of the FUV and NUV long-term trends – the residuals between mean 0 s $1\times1$ summed dark levels in each read port and the dark model (as defined at launch) – are plotted *versus* time as symbols. The current long-term trend models are overplotted as lines; they fit the data well and reduce the errors.

The exact nature and cause of the long-term trend is uncertain, and thus the exact form of the trend has evolved over time, as more data become available to characterize it. It is currently modeled by the sum of two sinusoids with periods $p_{Lj}$ and $p_{Lj}$/2, plus a weakly quadratic background. The variations on a timescale of $p_{Lj}$ are typically $\leq \pm 1$ DN (except for FUV port 3, where it is ±2 DN), on a background that has risen $\approx 4.5$ DN (FUV) or 0.5 DN (NUV). Hints about the origin of the trend may be found in the facts that it is independent of summing and $t_{\mathrm{int}}$, and that all the $p_{Lj} \approx $ 1 year; hence some yearly and biennial orbital variations (possibly due to seasonal temperature changes) may be playing a role. The full form of the long-term trend model $L_{j}$ is currently

3 $$ L_{j} = d_{j} \sin \bigl(2 \pi (t/p_{Lj} + \phi _{1j})\bigr) + e_{j} \sin \bigl(2 \pi (2 t/ p_{Lj} + \phi _{2j})\bigr) + f_{j} + g_{j} t + h_{j} t^{2} , $$

where $d_{j}$, $e_{j}$, $f_{j}$, $g_{j}$, $h_{j}$, $p_{Lj}$, $\phi _{1j}$, and $\phi _{2j}$ are constants, fit for each port using the offsets, over time, between the predicted and the actual average (cleaned) dark levels. The $h_{j}$ are considerably smaller for the NUV/slit-jaw imager (SJI) ports and are set to zero before a fixed time (which is different for the FUV and NUV/SJI CCDs) and return to zero at a second fixed time (identical for FUV and NUV/SJI). In the time interval early in the mission lacking darks (October–December 2013), we used measurements of the overscan lines (virtual lines created by extra CCD clock cycles) to guide the fitting. Empirically, the average 0 s dark level tends to track at or just above the average overscan values. The set of modeled $L_{j}$ compared with monthly dark data (as of June 2017) is shown in Figure 1. The root-mean-squares (RMS) of the trend fits with the data are satisfyingly small: the standard deviation of the model fit to the data for $1\times 1$ summing and $t_{\mathrm{int}} = 0$ s is $\sigma \approx $ 0.12 DN for the FUV ports (except for FUV port 3, where $\sigma \approx 0.28$ DN). The corresponding levels for the NUV/SJI ports show an average $\langle \sigma \rangle \approx 0.09$ DN.

Based on 4595 $t_{\mathrm{int}}$ = 0 s images from January 2014 through November 2015, the median offset error of the model for the lower binning modes ($n_{x} n_{y} \leq 4$) is < 0.3 DN for all FUV ports and < 0.1 DN for all NUV/SJI ports. The difference between the FUV and NUV/SJI ports is consistent with the factor 3 higher gain of the FUV CCD camera amplifier (Section 8.3). Considering all binning modes, the average offset error for a binning mode is always $<3$ DN, with RMS scatter $\sigma <$ 4 DN (FUV errors are slightly larger on average than NUV/SJI errors). Errors for longer integrations are discussed in De Pontieu *et al.* (2014).

The dark model is generated by iris_make_dark.pro, which in turn calls iris_dark_trend_fix.pro to generate the $L_{j}$. The model requires an hour of temperature data, which is generated externally. When housekeeping data are unavailable due to data loss, we search for an hour of valid temperature data $\pm nP_{ \mathrm{orb}}$ in time, where $n$ is an integer, and $P_{\mathrm{orb}}$ is the satellite orbital period. As long as $n$ is small, this retains the orbital phasing of the temperatures, and closely approximates the actual (missing) $T_{\mathrm{CCD}}$ and $T_{\mathrm{CEB}}$.

All the above described improvements to the dark correction were applied during the reprocessing of the IRIS Level 2 data archive between May and August 2017. Nevertheless, the calibration of IRIS data is an ongoing effort with gradual improvements introduced with time. Users are encouraged to always use the latest version of the Level 2 data.

### Hot Pixels

In addition to helping develop and refine the dark model, the dark observations provide a good dataset to observe the change in hot pixels. Hot pixels are defined as pixels whose values are above the local background average (typically $5\sigma $) for an extended period, and therefore falsely report an inflated value. We use this information to determine which pixels are hot, and track the change in the number of hot pixels over time.

**Figure 2:**
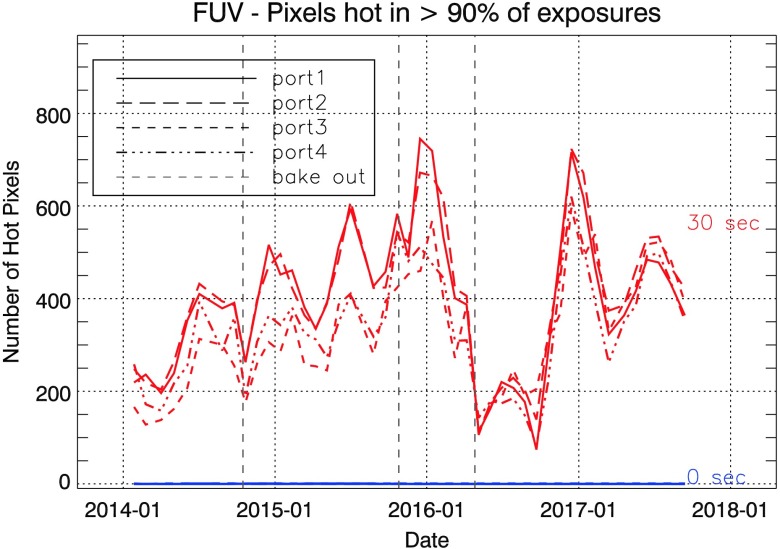
Plots showing the trend in the number of FUV hot pixels over time at the $>90$% threshold (NUV/SJI trends are similar, with 100 – 200 fewer $n_{\mathrm{hot}}$). The *vertical dashed lines* indicate the times of CCD bakeouts. The ports are shown separately, but follow a similar trend.

Each month, the short ($\approx 0$ s) and long ($\approx 30$ s) $1\times1$ summed integrations are separately grouped; each port is considered separately throughout. A first pass through the data is made, pixels $>5\sigma $ removed, and the median determined. Hot pixels are now redetermined using the “cleaned” median as a base level. We monitor the pixels that are “hot” in at least 10%, 50%, and 90% of the images during a month (typically covering a time span of two orbits); only the 50% and 90% pixels persist enough to be truly be considered hot. Examples of the number of hot pixels, $n_{\mathrm{hot}}$, over time for the FUV are shown in Figure 2. These plots are kept up to date at http://iris.lmsal.com/health-safety/longtermtrending/in_other.html.

The plot shows that $n_{\mathrm{hot}}$ is similar in all ports, with FUV ports showing 100-200 more hot pixels than NUV/SJI. There is considerable time variation, with a small average increase until the long CCD bakeout in April 2016, which appears to have reset $\approx 2/3$ of them. Shorter CCD bakeouts show weaker ($\approx 20$%), though still noticeable, reductions in $n_{ \mathrm{hot}}$. The variation in $n_{\mathrm{hot}}$ correlates with seasonal variations in $T_{\mathrm{CCD}}$, as the elevated dark current of hot pixels is highly temperature sensitive.

Investigation of the longer term behavior of individual hot pixels shows that they typically do not stay hot for long – most return to normal in a month or less. This is reflected already in the significant differences in $n_{\mathrm{hot}}$ at the 50% and 90% thresholds within a month; on average, $n_{\mathrm{hot}}(50\%)/n_{\mathrm{hot}}(90\%) \approx 1.6$. Typically only 7 – 8% (FUV) and 8 – 10% (NUV/SJI) of the pixels that were hot at the >50% short-term threshold were also hot for $>50$% of the mission months. There also appears to be a local maximum in hot-pixel number in December–January, coinciding with eclipse season.

### Future Improvements

In addition to regular recalibrations of the long-term trend, we are exploring methods for correcting hot pixels. At least a few of them, including some of the more prominent hot pixels, are persistent and show a good correlation with $T_{\mathrm{CCD}}$. Specifically, these potentially correctible hot pixels, $k$, show a dark current rate $\propto \exp(a_{j} + b_{j} (T_{\mathrm{CCD}j} + \delta T_{ \mathrm{CCD}k} ))$, where $\delta T_{\mathrm{CCD}k}$ reflects the incrementally enhanced temperature sensitivity of that pixel. As noted above, however, it is likely that only a few ($<10$%) will be correctable; furthermore, since for most hot pixels, $\delta T_{ \mathrm{CCD}k}$ eventually decays in time, the corrections would be imprecise for exposures that are temporally distant from the monthly dark datasets.

## Scattered Light and FUV Background

The IRIS FUV spectrograph (SG) experiences a modest level of infrared (IR) and visible (V) parasitic light. This was discovered during the course of in-flight calibration and commissioning. This parasitic light manifests as a fairly uniform background level that is present in the FUV short- and long-wavelength channels. Although the level is fairly uniform and lower than the intensity in the lines, this background interferes with flat-field determination and correction and must be removed. In this section, we outline the properties of the background, and methods for its characterization and removal. The NUV SG and NUV slit-jaw channels may also have a background contribution, but it is not as significant relative to the solar signal in these channels. This is no longer the case for the FUV slit-jaw channels. The decrease in FUV sensitivity, combined with an unchanged level of parasitic light, has recently led to noticeable ghosts in off-limb FUV images. While this ghosting is mostly cosmetic in nature, the removal of the FUV slit-jaw background may nevertheless be a topic of future work. The following paragraphs focus on the FUV SG background, since it potentially affects the quantitative analysis of spectra.

### Background Properties

Although the IRIS telescope rejects most of the incoming solar IR–V, a small portion is passed to the entrance of the spectrograph and slit-jaw imager. Imperfections in the light trap around the slit prism allow some residual light to scatter into the spectrograph. A second contributor is light that is first reflected off the slit-jaws toward the slit-jaw imager and then back to the slit-prism by one of the NUV filters in the filter wheel. Most of this light is reflected back out through the telescope, but a residual fraction enters the spectrograph again through imperfections in the light trap around the slit. The second component is only present if the filter wheel is configured for NUV slit-jaw imaging; the FUV background is reduced when the filter wheel is configured for FUV imaging.

The background component is most evident in the FUV channels because there is little continuum intensity at these wavelengths. The background does seem to be present in the NUV channel as well, but it is more difficult to separate from the bright and highly structured spectrum around the Mg ii lines. It is also less prominent because of the lower gain setting of the NUV camera amplifier.

**Figure 3:**
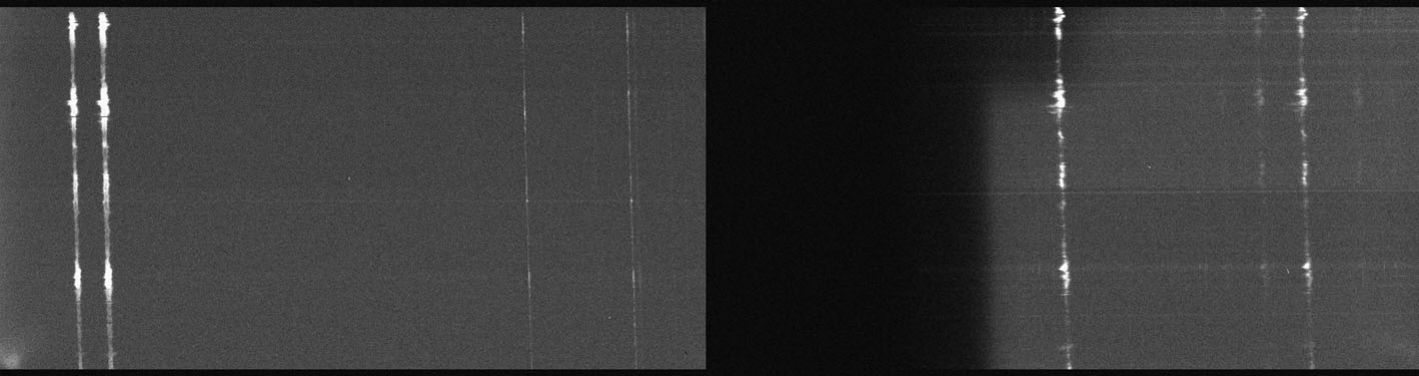
Typical long exposure from the FUV-S CCD on the *left* and the FUV-L CCD on the *right* showing the scattered light background. The *dark area* on the short-wavelength side of the FUV-L detector is not illuminated by the spectrograph optics.

A full-frame, long-exposure image from the two FUV CCDs is shown in Figure 3. The background in the FUV channels is fairly uniform across the spectrum, with the exception of the edge on the blue side of the FUV-L spectrum and a corner that overlaps the brighter Si iv line near the top. The slit in the FUV-S channel does not extend over the full CCD area, and the region at the top of the detector contains background, but not spectrum. This fortunate alignment offset can be used to determine the background level accurately for on-disk pointings.

**Figure 4:**
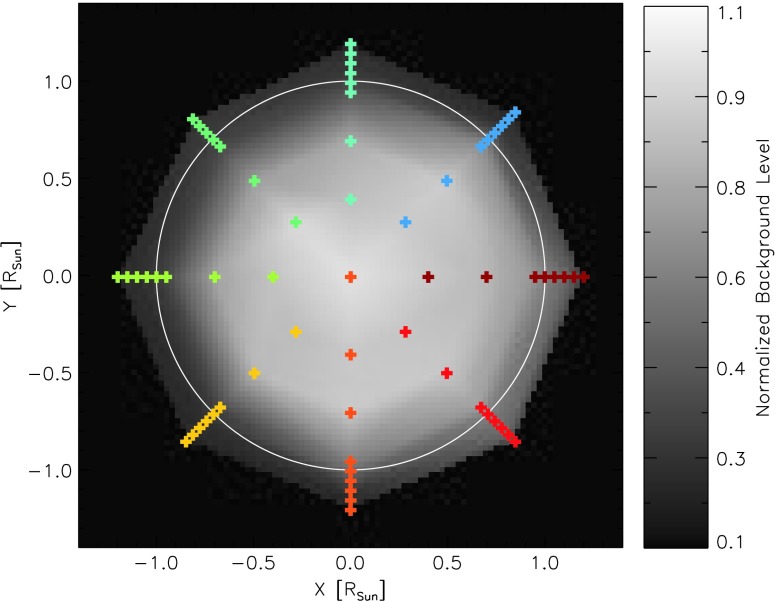
Color-coded pointing positions on a coarsely interpolated map of the background intensity.

**Figure 5:**
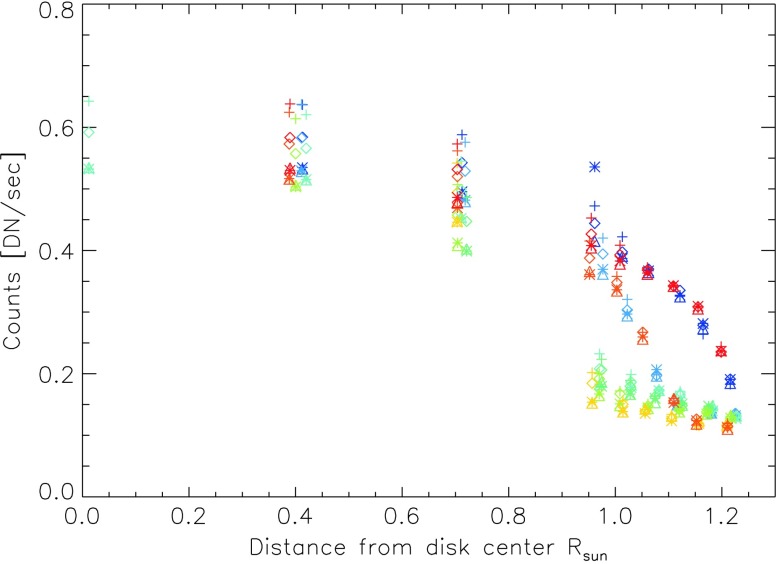
Background intensity as a function of solar radius with the same color-coding as used in Figure 4.

While the spatial pattern of the background on the CCD changes relatively little with pointing, the total intensity of the background changes with the IRIS pointing by a factor of about five from the solar center to the limb. The peak intensity is slightly offset south and west from Sun center, and the intensity as a function of radius from this center has the appearance of a smoothed or defocused limb-darkening function. The orientation of the pointing dependence of the stray-light pattern is fixed with respect to the telescope orientation, and has the same orientation with respect to the telescope during rolled observations. Figure 4 shows the intensity pattern interpolated from observations at the plotted positions for $\text{roll} = 0^{\circ }$. In Figure 5 it is clear that the background intensity is offset from disk center, but that the pattern shows a regular behavior as a function of radius from this offset center.

For the reasons stated above, the background intensity is higher when an NUV filter is in place in the SJI at the time of the observation. The background pattern, however, remains largely the same. Figure 5 shows the average intensity of the background as a function of radius. The different filters are indicated by triangles (1330 Å), plus signs (2796 Å), stars (1400 Å), and diamonds (2832 Å).

### Characterization

Observations for characterization of the FUV background are taken on a monthly basis outside of eclipse season. They consist of 30 s exposures with the FUV CCDs at each science filter position (1330 Å, 1400 Å, 2796 Å, and 2832 Å) along eight radial spokes covering the solar disk and limb (see Figure 4). Dark frames of the same exposure length are also taken at every position.

The observations are processed to remove cosmic ray spikes, and the darks are subtracted from the light frames. The median of the FUV background is taken from the region above the edge of the slit at the top of the FUV-S image. Each image is normalized to this median, and the average is taken across all filters for all of the off-limb pointings to create the average background frame. The frame should contain only the background and should have no spectral lines.

Further analysis is done for the set of background intensity values from each filter to determine the center of the intensity distribution and its behavior as a function of radius from this center. The solar $X$-coordinate of the slit and solar $Y$-coordinate at the center of the slit are paired with the background intensity values and fitted with a smoothed limb darkening function. We adopt a limb darkening function of the form

4 $$ I(\theta )/I(\theta =0) = 1 - u - v + u \cos {\theta } + v \cos {^{2} \theta }, $$

where $u=0.97$ and $v=-0.22$ are the tabulated values for the limb-darkening function at 5000 Å from Section 14.7 of Allen’s Astrophysical Quantities (Cox, 2002). The free parameters in the fit of the limb darkening are $X_{0}$ and $Y_{0}$, the middle of the background distribution in solar coordinates, $dw$, the width of a Gaussian profile used to smooth the limb-darkening function, $a_{0}$, the offset of the base of the limb-darkening function, and $a_{1}$, the amplitude of the limb-darkening function from the base.

**Figure 6:**
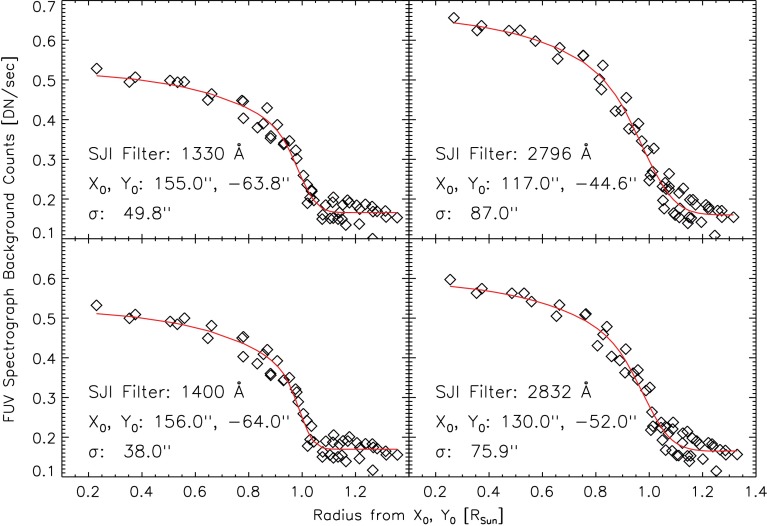
Median level of the FUV background as a function of radius from position $X_{0}$, $Y_{0}$ for each filter wheel position used for science observations. The fit in *red* is a broad-band limb-darkening function smoothed with a Gaussian.

Figure 6 shows the results of fitting the limb-darkening function for all four filters from the same set of observations. The NUV 2796 Å filter generates the highest background level, while the 2832 Å filter is slightly decreased, and the centers of the distribution are significantly different. The FUV 1330 and 1400 Å filters show almost identical background properties with the lowest level.

### Removal

The IR–V background is removed from the FUV SG frames of science observations during reduction from a Level 1 to Level 2 data product. The background calibration results nearest in time are used to apply the correction. Based on the filter and pointing of the observation relative to the orientation of IRIS, the median background level is calculated using the empirical, smoothed limb-darkening function and applied to the median background frame produced during the calibration. This scaled image is then subtracted from the science image.

The implemented background correction uses pointing information to adjust the intensity of the background, but not its shape. In reality, however, the shape of the background in the FUV-L channel changes slightly with pointing, especially in the vicinity of the Si iv 1394 Å line. Even though these changes are small compared to the typical Si iv line intensity, it is unwise to use the region close to, and longward of, the Si iv 1394 Å line to determine the FUV continuum level. The FUV spectrum shortward (blueward) of 1394 Å is less affected and better suited for continuum level measurements.

### Trends

The properties of the FUV background have changed slightly over the lifetime of the IRIS mission. The center of the limb-darkening function has drifted slightly with time, while the smoothing width that determines the shape of the limb-darkening function is fairly constant for each filter. The greatest changes are in the intensity amplitude and pedestal for the limb-darkening function, which show consistent annual variations as well as significant long-term drifts. The pedestal level has steadily decreased, while the amplitude of the limb darkening function has slightly increased. All filters show nearly the same pedestal level. This may indicate that the pedestal level is caused by general scattering of light off optical surfaces inside the instrument.

## Flat Field

Raw images from IRIS contain a variety of undesirable features that need to be removed before data analysis. These include the pattern of gain variation on the CCD, dust on various optical surfaces near focal planes in the spectrograph and imagers, and possible vignetting. Spectrograph images also contain a fixed intensity variation pattern along the spatial dimension that is due to slight imperfections in the slit. In this section we describe the techniques used to construct flat fields from in-flight data.

### Slit-Jaw Imager Flat Fields

#### Description

The flat-field method put forth by Chae (2004) can be used to build a flat field from a set of non-uniform images where the illumination pattern has been shifted relative to the detector in each image. The technique extracts the illumination pattern (or object) from the flat-field pattern using a least-squares method, keeping the object shifts and illumination level as free parameters. This technique has been proven more robust than other similar methods (*e.g.* Kuhn, Lin, and Loranz, 1991).

#### In-flight Observations

Flat-field observations for the SJI are taken on a monthly basis using observations of quiet-Sun or network regions, which are less likely to change during the eight-minute observing sequence. The field of view must not include the solar limb; regions close to disk center are preferable because they will not include large systematic gradients that are due to limb darkening or brightening. Prior to April 2016, three sets of observations from different regions of the Sun were taken for all filters and combined in processing to reduce the effect of noise and residual solar structure in the individual flat fields. The number of sets was increased from three to six in April 2016 for the FUV filters to compensate for sensitivity loss at those wavelengths.

**Figure 7:**
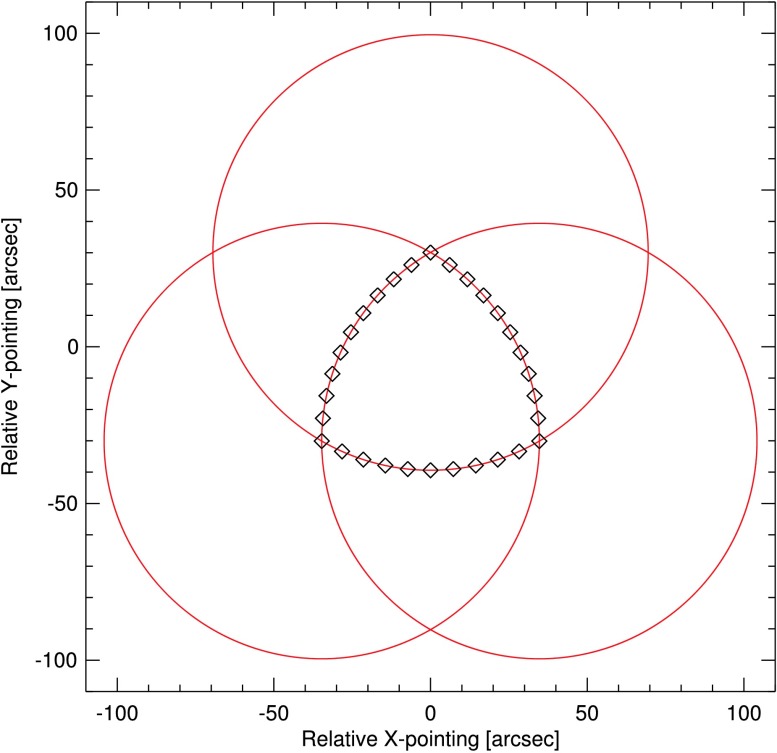
Example of a Reuleaux dither pattern, as used for the SJI NUV flat-field observations, with 30 pointings and a pattern size of 70 arcsec.

The images for each filter wheel position are taken in rapid sequence to minimize the amount of change in the solar scene. During the sequence, the pointing is dithered about the center of the field in a Reuleaux triangle, as suggested by Chae (2004). This is a commonly used dither pattern in optical and radio astronomy, which is formed from the intersection of three circles of equal radius. The dither pattern used for the IRIS SJI NUV flat field observations is shown in Figure 7. A pattern size of 70 arcsec was adopted for the NUV channels to properly sample the large shadow from the Šolc filter mask (Berger *et al.*, 2012) that appears in the upper right quadrant of the images. The sequences for the 1330, 1400, and 2796 Å filters are taken with the telescope out of focus to decrease the contrast of dynamic features and spread intensity more evenly over the detector. The sequences for the 2832 Å filter remain in focus as the solar granulation seen at 2832 Å is sufficiently stable over the duration of the flat-field sequence.

#### Calibration Processing and Products

The software to produce the flats for the SJI read the data, despike, construct, and subtract dark images, and provide a wrapper for the Chae code. After flat fields for the FUV and NUV sequences have been produced, the slit is removed and the images are averaged together. Removing the slit from the flats allows the slit to remain visible in science images after flat-fielding. The slit removal treats the pixels around the slit as missing data and smoothes the large-scale pattern over the slit. That region is then multiplied by a pre-flight flat (created from UV lamp exposures) to re-establish the small-scale pattern. The flats are normalized to the average signal level in the observations.

The flat fields are provided as FITS files to the data-processing pipeline. We provide images for each filter both with and without the slit. Files from each month are monitored for quality. In iris_prep, the nearest flat available at the time of processing is applied.

#### Results

**Figure 8:**
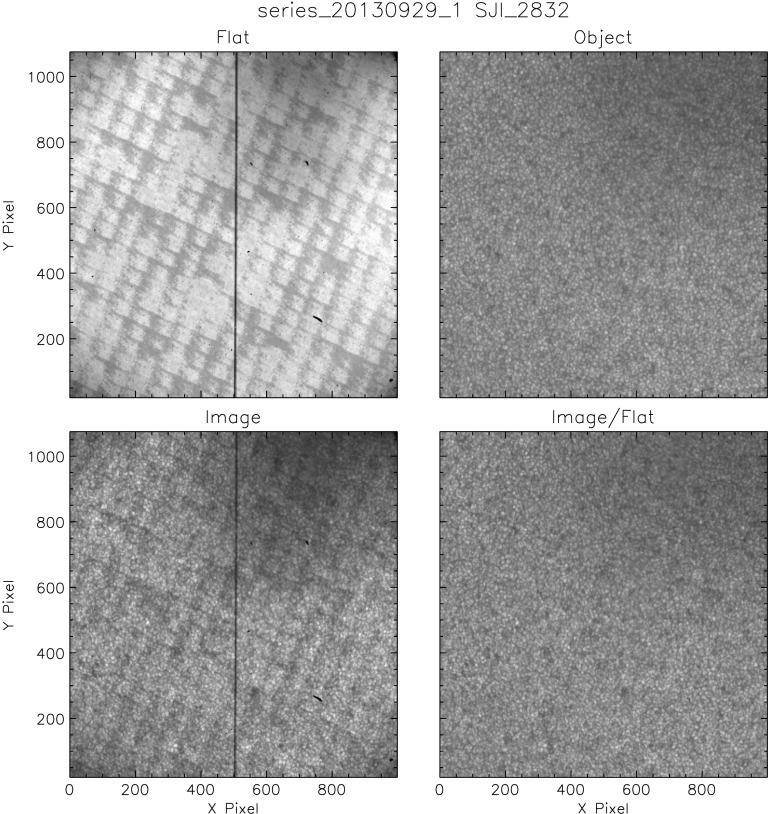
Example of the resulting flat field and object frame from Chae’s method applied to the 2832 Å channel of the SJI. The *two bottom frames* show an original image from the observed sequence and its correction by the flat field.

Figure 8 shows an NUV flat, the object image, and a quiet-Sun image before and after flat-fielding. Flat-fielding clearly improves the quality of the image. The characteristic small-scale anneal pattern of the e2v CCD, visible in both the flat and the raw image, is no longer present in the corrected image. The vignetting from the Šolc filter mask in the upper right corner is also much reduced. Nevertheless, the NUV object image still shows a significant residual from the Šolc filter mask, which indicates that the correction is not perfect. Chae’s technique assigns about half of the darkening to the object frame, and the flat field only corrects for part of the drop in intensity due to the mask. A larger dither pattern might help the technique to distinguish this pattern, but the duration of the flat-field sequence would become excessively long. The flat-field correction away from the upper right corner is quite good, and the other corners do not seem to show vignetting at all.

**Figure 9:**
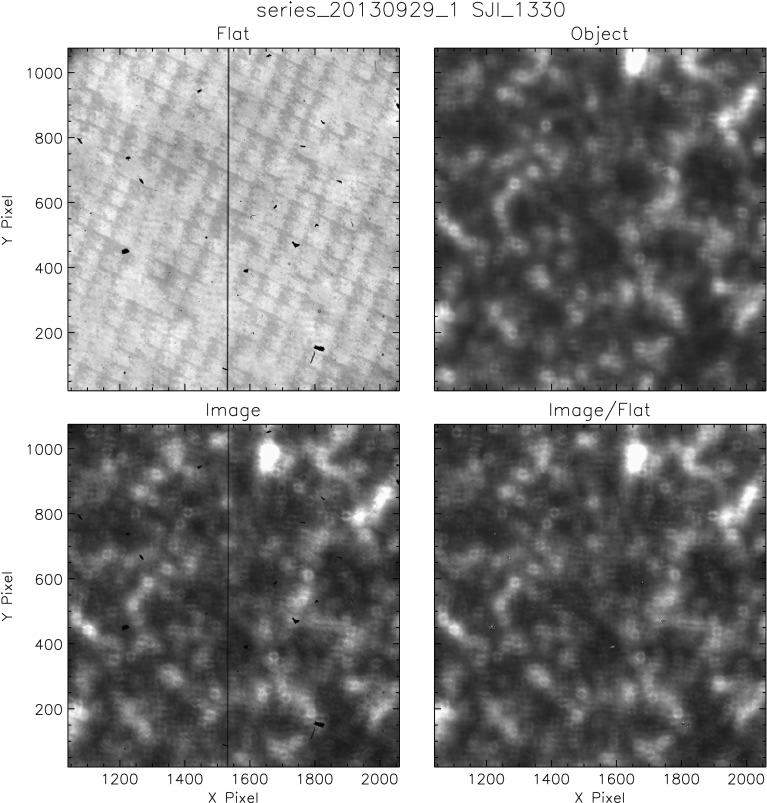
Example of the resulting flat field and object frame from Chae’s method applied to the 1330 Å channel of the SJI. The *two bottom frames* show an original image from the observed sequence and its correction by the flat field.

Flat-fielding is also successful with FUV images, even though it has its own challenges. Because the contrast in the FUV object is high and slightly changes with time, the flat fields from Chae’s method contain some residual structure (Figure 9). To correct for this, multiple sequences are processed using Chae’s method, which are then averaged together to even out the structure in the final flat field.

**Figure 10:**
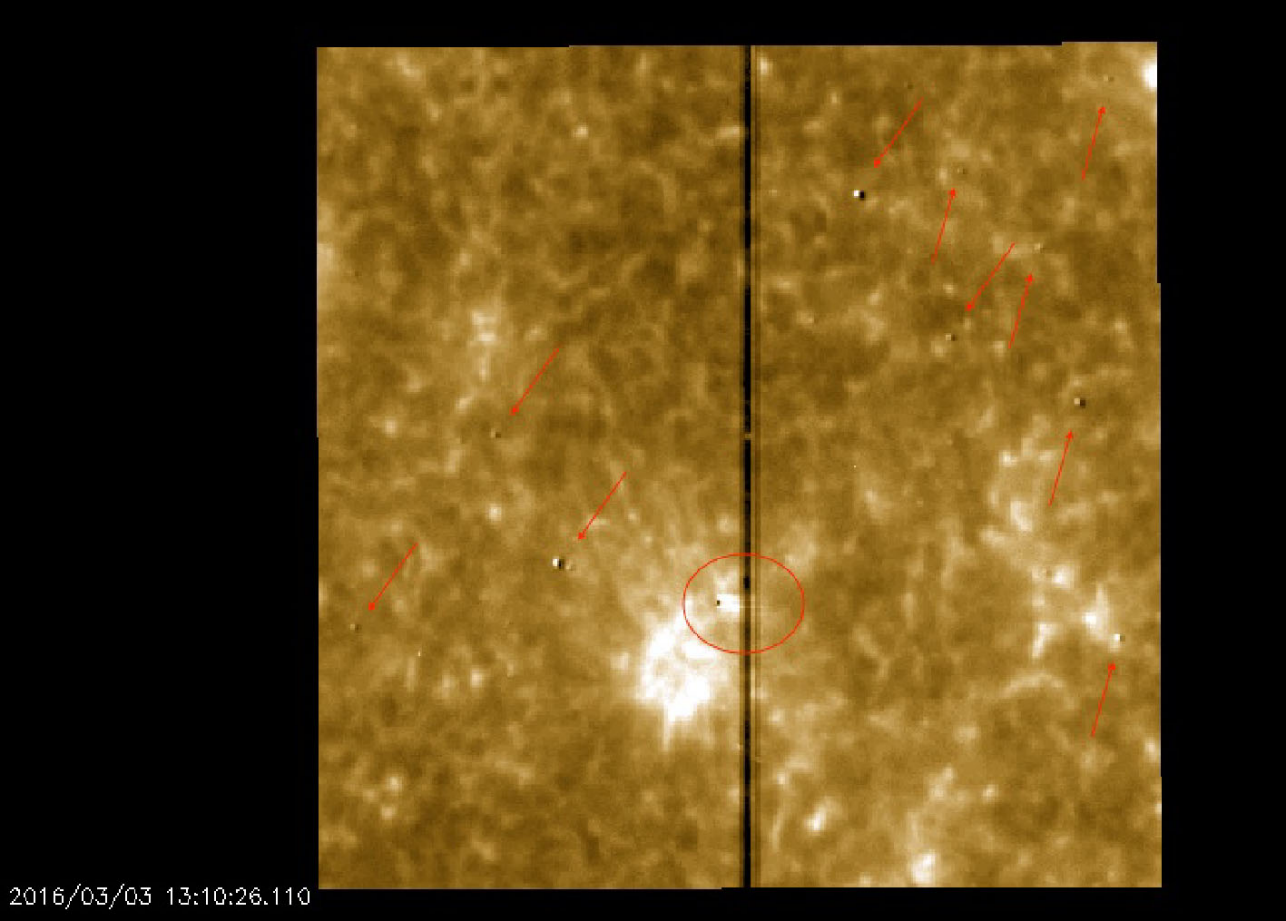
Level 2 data from the 2796 Å channel of the SJI. The slit prism is shifted in the science observation with respect to the flat field so that dust specks on the surface of the slit prism show bipolar signatures that are indicated by the arrows. The removal of the slit in the vicinity of a dark dust speck on the detector produces a streak across the slit (*circled*).

Finally, small dust specks on the slit-jaws may introduce subtle artifacts. Residual filter wheel position variations and orbital temperature changes may cause the detector image of these dust specks to shift by up to a few pixels (mostly in the $x$ dimension). If the shift between the flats and the science images is different, artifacts result that look like bipoles in the processed data. The position of the dust specks is well known so they can easily be distinguished from real solar features. An example of these flat-field residuals in the NUV channel is shown in Figure 10.

#### Dosage Burn-in

**Figure 11:**
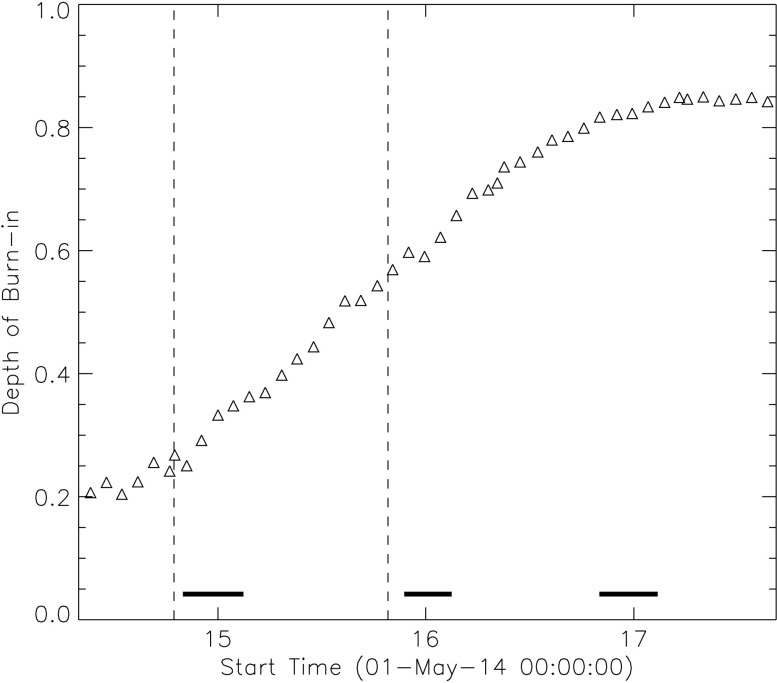
Depth of the 2D Gaussian fitted in the flat field of each month. This is the depth relative to the “continuum” fit for the Gaussian, which translates into a measure of the steepening of the profile. The *horizontal bars at the bottom of the plot* indicate the eclipse seasons. The *vertical dashed lines* indicate detector bakeouts.

Dosage burn-in has been contributing to a loss of sensitivity in the FUV channels of the slit-jaw imager. The burn-in preferentially affects the middle of the detector more than the edges, so there has been a gradual deepening of the burn-in pattern, which is fairly smooth and Gaussian. Very little change is apparent in the NUV channels. Figure 11 shows the characterization of that depression with time. Dosage burn-in in the slit-jaw images is mostly corrected by the flat field, although there remains a residual component similar to the one caused by the Šolc filter mask in the NUV images.

#### Slit Brightening

**Figure 12:**
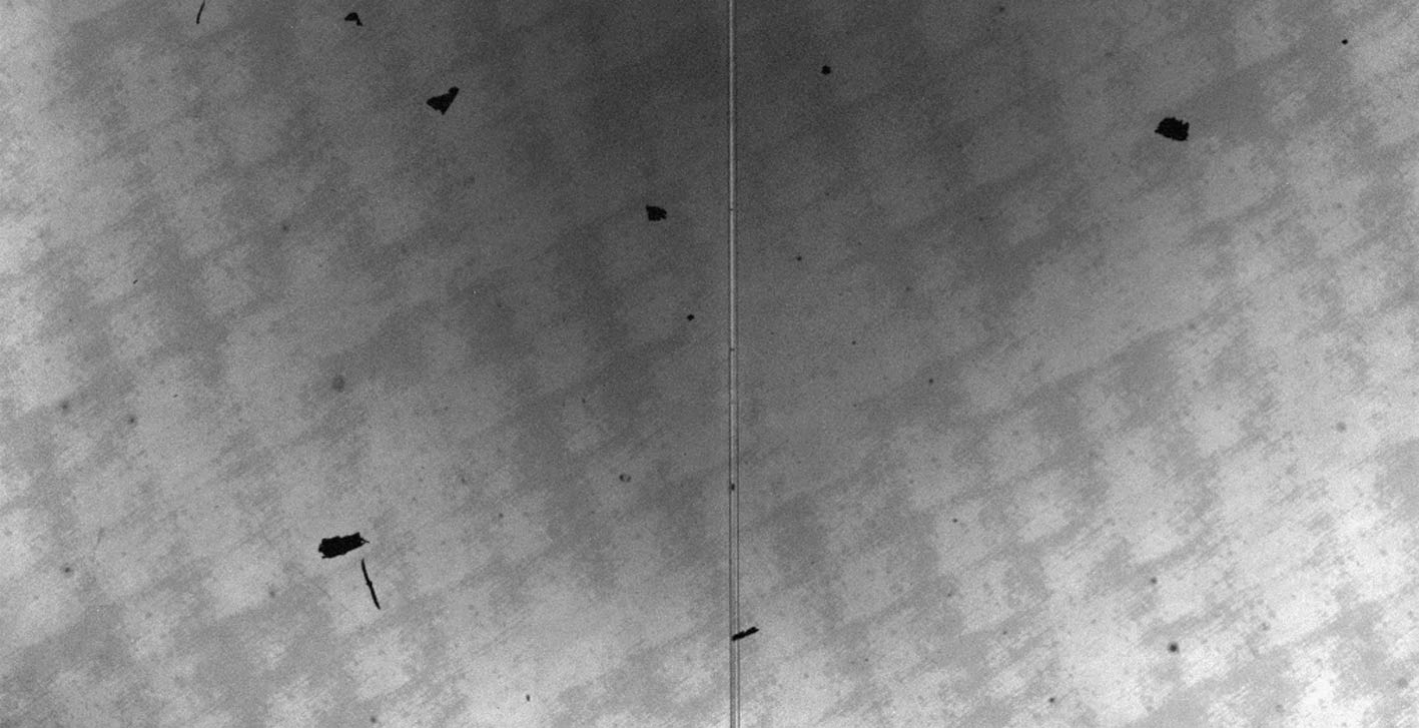
Lower half of the 1330 Å flat from January 2016. This is a raw flat, *i.e.* the slit was left untouched. In some places, the slit has become brighter than the surrounding areas. Dark dust specks on the slit remain dark. Dosage burn-in is visible as a large-scale pattern.

The apparent intensity of the slit as seen in the 1330 and 1400 Å slit-jaw images has been increasing with time since launch. The effect is largest and most noticeable in the 1330 Å channel, where the slit at the middle of the detector became brighter than the region surrounding the slit in the Fall of 2015. Figure 12 illustrates the bright slit in a raw flat field, but it is equally seen in regular SJI images. The slit brightening makes automated detection of the fiducial marks in the slit less reliable, which has an impact on some spatial calibration tasks.

The cause of the slit brightening is not understood. We know that it is not an artifact of dosage burn-in on the detector, otherwise it would prominently appear in exposures with the onboard calibration LED. Instead, the root cause must be at the location of the slit, which receives a considerable dose of FUV radiation on orbit. The slit-jaw images were created by deposition of chromium and aluminum on the $\mathrm{MgF _{2}}$ slit prism. The metal layers and the slit then received a $\mathrm{MgF_{2}}$ overcoat to maximize the FUV reflectivity. We speculate that either deterioration of the slit-jaw coating reflectivity (relative to the residual reflectivity of the uncoated $\mathrm{MgF _{2}}$ surface in the slit), effects of deposited contaminants, or a combination of both may have caused the observed slit brightening.

### Spectrograph Flat Fields

**Figure 13:**
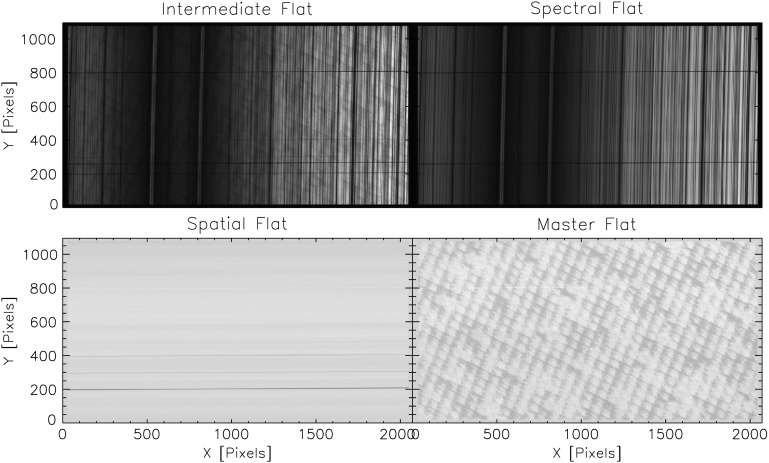
NUV spectrograph flat fields. See text for details. The two prominent *horizontal lines in the top panels* are caused by the fiducial marks in the slit. The spatial flat (*bottom left panel*) has the fiducial marks removed; the remaining *dark horizontal line near the bottom* is caused by a small dust particle on the slit.

There are several artifacts in IRIS raw spectra that can in principle be removed with a flat-field process. These artifacts include dust particles in the spectrograph slit, spectrograph vignetting, the CCD anneal pattern (see, *e.g.*, the lower right panel of Figure 13), and detector burn-in from high doses of UV exposure. We have conducted several types of flat-field-related calibrations prior to launch. In particular, we acquired CCD flat-field images at several UV wavelengths, although not at all of our observing wavelengths. However, we found that the CCD anneal pattern is very similar over a range of wavelengths, except for the amplitude. We have recreated fairly accurate CCD flats by appropriately scaling the amplitude of the anneal pattern from flats at different UV wavelengths. We also confirmed that vignetting in the spectral direction is negligible; response variations are dominated by the spectral response of the optical components and coatings. The spectrograph spectral response is discussed in Section 8.

On-orbit spectrograph flat fields are more difficult to produce than SJI flat fields, because IRIS neither has a UV calibration source, nor can it move the solar spectrum across the detector. The general approach for IRIS is as follows: i) Create a raw flat field by averaging a large number of frames taken at many different quiet-Sun locations. ii) Produce a spatially smoothed “spectral flat”. iii) Produce a spectrally averaged “spatial flat”. iv) Derive a detector flat by dividing the raw flat by the spectral flat. This essentially removes the solar spectrum. v) Divide the detector flat by the spatial flat. This separates variations along the slit from the detector flat and allows them to be applied separately. The final detector flat field contains the CCD anneal pattern, but is blind to detector burn-in and vignetting in the spectral direction.

We are successfully applying the above approach to NUV SG data. However, the approach was not successful in the FUV. By their nature, FUV spectra show extreme spectral and spatial variations that lead to high levels of noise in the flat fields. Instead, we flat-field our FUV SG data only with a pre-launch detector flat. The following paragraphs discuss the NUV flat-field process in more detail.

#### In-flight Observations

The spectrograph flat-field observations are conducted on a monthly basis. Both NUV and FUV flat-field data are acquired, even though only the NUV data are currently used for flat-fielding. The observations should ideally be confined to quiet-Sun regions near disk center, but the quiet Sun does not provide enough counts in the FUV channels, so network or a quiescent active region plage is used. Thirty-second exposures are taken with the telescope out of focus, while the pointing is semi-randomly slewed about the center of the field to further average over the solar structure. In addition, the slit is rastered. 150 images are taken, and darks are taken every 10 images so that the dark correction will be accurate for the flat field.

#### Calibration Processing and Products

Images from a flat-field sequence are dark subtracted using the darks taken with the observation if they are available and using the iris_prep dark correction if they are not. The data are despiked. Then all the images are averaged together to produce the intermediate flat field. The intermediate flat is further processed into a detector flat and a spatial flat as described earlier in this section. The fiducial marks are removed from the spatial flat through interpolation. This has the effect that the fiducial marks remain in the data processed by the flat field.

The spatial flat is retained separately, so that it can be shifted and applied to compensate for thermal drifts between slit and detector. This compensation is not currently implemented, partly because the drift is quite small and partly because the prerequisite detection of the fiducial mark location is not sufficiently reliable.

For the NUV flat field applied in the processing pipeline, the detector flats from several months are averaged together and the nearest spatial flat is used. For the FUV we use only the pre-flight lamp flat, but the FUV flat data are retained for the record.

#### NUV Results

Figure 13 shows the results of this technique applied to the NUV data. The top left panel shows the intermediate flat field, which is an average of a sequence of 150 images. The top right shows the final spectral flat field, and the bottom left shows the final spatial flat field without the fiducial marks. The bottom right panel shows the result of removing all the spatial and spectral features from the intermediate flat field. The master flat field is similar to the NUV lamp flat field, but it has a slightly different contrast and contains some very subtle residuals of solar features.

### Spectrograph Detector Burn-in

Changes in sensitivity of the CCD, or charge burn-in, is mainly a concern for the bright C ii and Si iv lines in the FUV. For the SJI, burn-in is not an issue, and can easily be measured and removed using the flat-fielding technique. For the spectrographs, burn-in might occur isotropically along the spatial dimension of a line, causing an apparent deepening in the emission line core with respect to the dimmer wings, but more likely, the damage will show some spatial variation, with most of the burn-in occurring at the middle of the slit, where bright interesting targets tend to be centered. Because spectral filtering is applied during the spectrograph flat field processing, the final flat field cannot properly account for burn-in.

**Figure 14:**
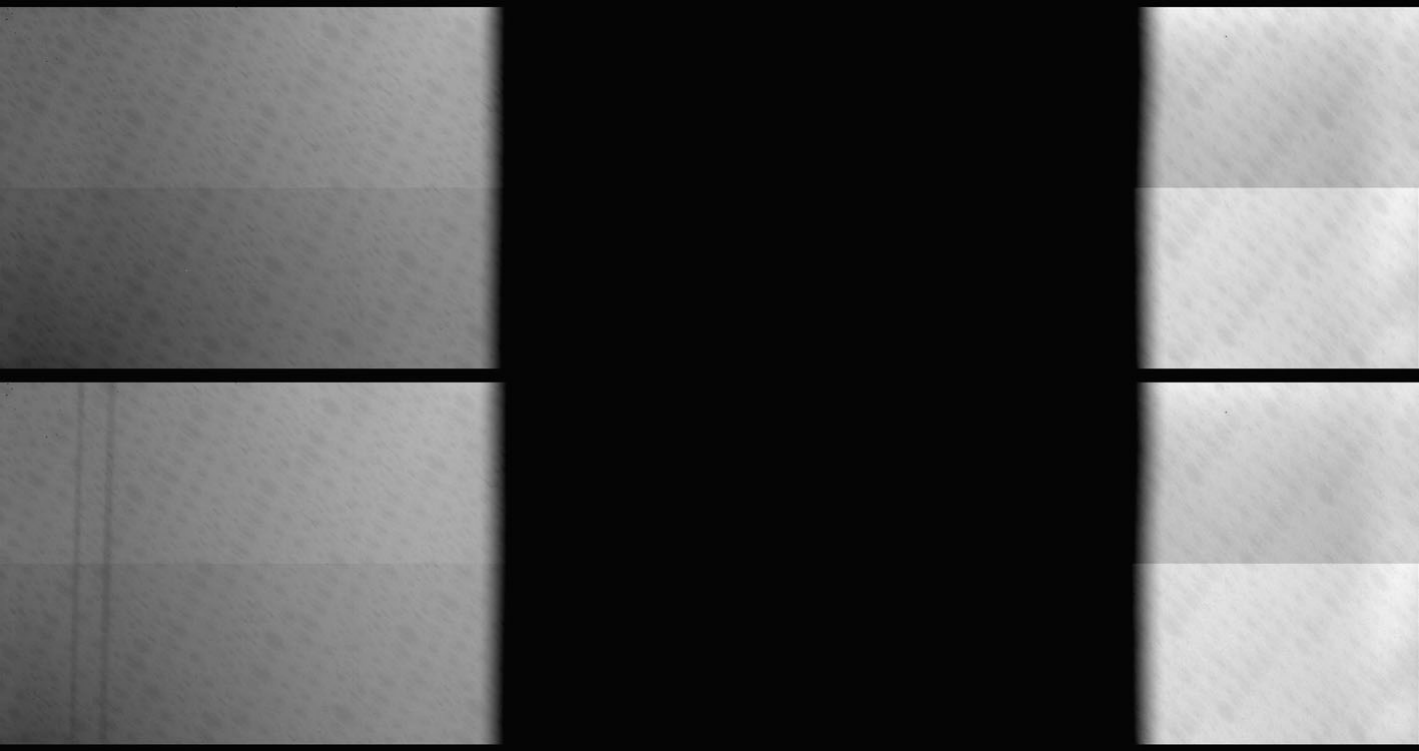
Blue LED images taken shortly after launch (*top*) and in October 2014 (*bottom*). The second image shows the burn-in pattern from the two C ii lines on the left. The LEDs have not been designed to fully cover the CCDs, hence the large non-illuminated area in the middle.

However, there are indications for detector burn-in in the FUV SG. IRIS occasionally acquires exposures with an onboard blue LED. IRIS carries LEDs primarily for verifying that the instrument is functional, so these LEDs have not been designed to fully illuminate all detectors. The FUV LED covers the locations of the two C ii lines and the 1403 Å Si iv line, but not the Si iv line at 1394 Å. The top panel of Figure 14 shows an LED exposure taken on 17 July 2013, just before IRIS first light, and the exposure in the bottom panel was taken on 4 October 2014. The latter clearly shows the location of the two C ii lines as bands with slightly lower sensitivity. The location of the Si iv line does not show a significant signature because the Si iv line is at least four times weaker and likely to cause much less burn-in.

**Figure 15:**
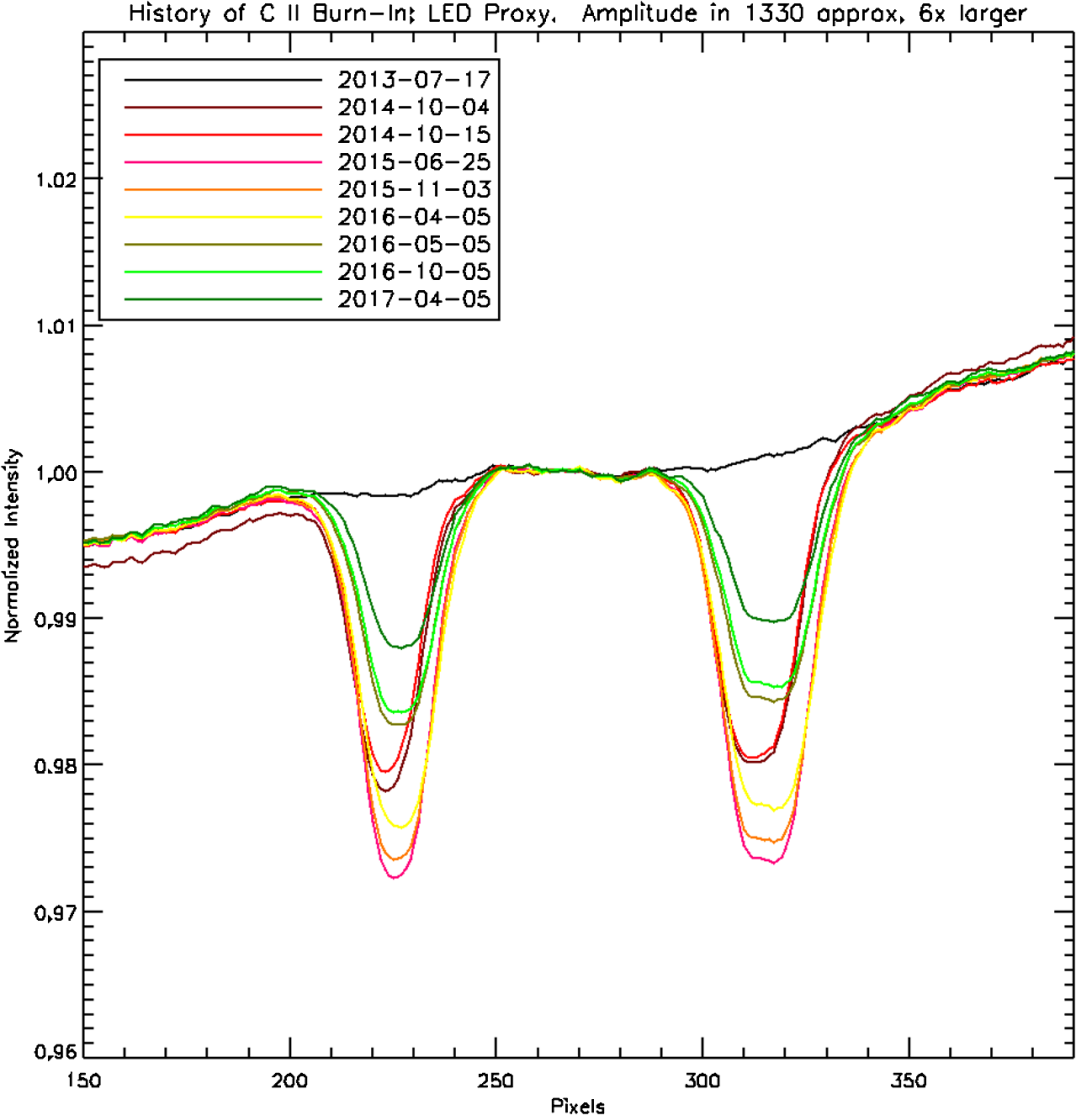
Burn-in profile from the C ii lines in selected blue LED images. The amplitude of the burn-in at the actual FUV wavelengths is about six times larger than it is at the blue wavelengths shown here.

If the burn-in in the LED images were similar to the burn-in in the FUV, then we could use the LED data as a proxy for the FUV burn-in. Figure 15 shows the C ii burn-in profile in selected LED images taken over the course of the mission. Each profile is an average along the slit. We find that the burn-in amplitude at the blue LED wavelength increased to about 2.8% in mid-2015 and decreased thereafter. Some of the decrease correlates with detector bake-outs, *e.g.* between April and May 2016, but most of it does not.

Measuring the burn-in with FUV light is more difficult since we cannot scan the solar spectrum across the detector. However, we found that we can move the spectrum by up to ten pixels over the course of an hour if we apply a thermal gradient across the spectrograph with the IRIS instrument heaters. If we assume that the average C ii line profile of a quiet-Sun region does not change over an hour, then we can use this spectral scan to probe the approximate depth of the burn-in at the nominal location of C ii. We have performed this calibration in March 2015 and again in June 2017. We found typical burn-in depths of about 16% in 2015 and about 5% in 2017. The results are accurate to only about 30%, probably because of the slowness and small range of the scan combined with residual small variations of the solar C ii profile. Nevertheless, the measurements indicate that the burn-in has decreased in the FUV as well, not only in the blue light of the LED. The relative decrease was comparable within the accuracy of the FUV measurements: a factor of about 3.2 in the FUV *versus* 2.5 in the blue over the same period. The test also suggests that the burn-in is about five to six times deeper in the FUV than in the blue.

These results indicate that the burn-in pattern in the blue LED images may be a good proxy for the FUV burn-in, if scaled by about a factor of five to six. As a sanity check, we compare the depth of burn-in in the FUV and in the blue with the depth of the CCD anneal pattern at those two wavelengths. We indeed find that the anneal pattern is very similar and the amplitude of the pattern is 6.0 times larger in the FUV. This is fully consistent with the ratios we found for the burn-in.

It is important to note that we are not correcting for any burn-in in the spectrographs at this time, although we may implement a correction in the future. Nevertheless, the burn-in should be taken into account if the C ii lines are being analyzed for subtle deviations from a Gaussian profile and for detailed line width work.

In contrast to the FUV SG at C ii, the detector of the NUV SG does not show a significant burn-in at the location of the Mg ii line cores in images taken with the blue LED. The lower energy NUV photons do not appear to have the damaging effect of the higher energy FUV photons.

## Geometric Correction

### Description

In order to facilitate scientific analysis, it is desirable to have spatial and dispersion directions in a spectrum aligned with the pixel dimensions in the observations. However, distortions from rectilinear dimensions are present in any spectrograph. In addition, the IRIS spectrograph design produces spectral lines that are inherently curved. The top panels in Figure 13 show an NUV spectrum before distortion correction.

### Calibration Approach

A spectrum from IRIS contains bright emission lines (for the FUV) or a combination of absorption and emission features (for the NUV) crossed by two dark fiducial marks, which are sections of the slit that have been intentionally coated as a spatial reference. The geometric calibration is determined by measuring the very features that should be rectilinear, the bright or dark spectral lines, and the dark slit fiducials crossing the spectrum. We assume that the warping is a smoothly varying field that can be characterized by a 2D second-order polynomial function. The transformation from warped to unwrapped coordinates can be expressed by

5 $$ x' = \sum_{ij} k_{x_{ij}} x^{j} y^{i}, \qquad y' = \sum _{ij} k_{y_{ij}} x^{j} y^{i},\quad 0 \leq i,j \leq 2, $$

where $k_{x_{ij}}$ and $k_{y_{ij}}$ are the to-be-determined coefficients of the warping function, the original image coordinates are $(x,y)$, and the transformed coordinates are $(x',y')$. For arbitrary features in the image, such as the crossings between spectral lines and fiducials, we need to know the location in terms of the original image coordinates, and where we wish all crossings of a particular line and all the crossings of a particular fiducial to be in the transformed image. Given many such $(x,y)$ and $(x',y')$ coordinate pairs, we can fit the coefficients $k_{x_{ij}}$ and $k_{y_{ij}}$ using a least-squares technique. Once we have the function, the task of determining the transformed coordinates of all of the pixels in the original image is trivial.

We call the 2D table of $x$ and $y$ coordinate transforms a “distortion map.” The distortion map is an array the size of the transformed image where each element contains the $x$ or $y$ coordinate of that pixel in terms of the original coordinate frame. Given a distortion map for both $x$ and $y$ coordinates, the intensity values of an image can be transformed from one coordinate frame to the other using a standard interpolation technique.

In the above process, we allow the spectral and fiducial lines to have arbitrary positions assuming only that they have a fixed separation, so it still remains to make the wavelength a linear function of pixel position. Combining a wavelength solution with the above process would require a higher-order polynomial function, which leads to greater uncertainty, and non-unique solutions, when the $(x,y)$ pairs are too sparsely sampled. Therefore we determine a wavelength solution after the distortion map is determined (and the spectral lines are aligned with the vertical columns in the image) and combine the result of the wavelength solution with just the distortion map for the $x$ coordinate, to linearize the wavelength. It is not the goal of this calibration to provide a definitive wavelength reference. That is discussed further in Section 6.

### Acquisition and Processing

The same data taken for flat fields are used to determine the in-flight geometric correction for the NUV SG. The NUV has many narrow absorption lines of neutral atomic species, which have higher contrast than the continuum, and they show small velocity perturbations, which can be averaged over in time-series observations of the flat field.

Good in-flight characterization is not possible for the FUV SG. The bright lines of the transition region are broad, often have high velocities with respect to the average central position of the line, and large systematic Doppler shifts. Some neutral lines appear in emission in the FUV channels, but they are often too dim to see across the entire field of view, even in heavily averaged spectra. There are also far fewer lines in a typical spectrum. All of these effects cause the geometric solution and the wavelength solution to be poorly constrained from in-orbit FUV data. Instead, we use pre-launch data to determine the FUV geometric and wavelength corrections.

**Figure 16:**
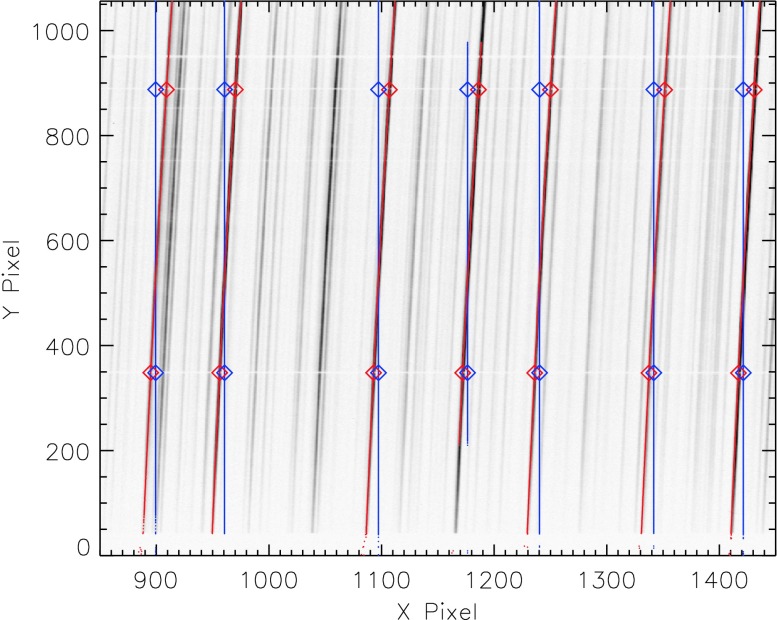
Portion of the deuterium lamp spectrum observed with the FUV-S spectrograph channel during ground testing. Fitted line positions and fiducial positions are shown in *red*, while the desired rectilinear coordinates are shown in *blue*. The fiducial/spectral line crossings are indicated by the *diamonds*, while the line centroids are plotted as *small points*. The 2D fitting of the *red* and *blue points* yields the geometric transformation. The image is upside down with respect to typical IRIS Level 1 data and is displayed in inverted grayscale to show emission lines as dark features.

During integration and testing, the FUV SG was illuminated with a deuterium lamp source, which has many narrow emission lines that are due to excitation of electronic transitions in molecular deuterium. An example of the deuterium spectrum seen with the FUV-S channel is shown in Figure 16. A quadratic fit was performed on the spectral lines with respect to the $y$ dimension of the image, and a linear fit was performed on the fiducial lines with respect to the $x$ direction. We found that the line and fiducial marks wander to different positions during tests at different temperatures, but the slope, shape, and position of the lines with respect to other lines in the spectrum change very little and amount to changes of 0.1 pixel over the full area of the detector. We concluded that the high-order terms in the distortion in the spectrograph images are stable to thermal variation and that only the offsets (discussed in Sections 6 and 7) change with temperature.

This robustness has led us to adopt static corrections for the geometric distortions for the FUV and NUV spectrographs. The averaged series of dark- and flat-corrected data from the NUV flat-field observation of 6 March 2014 was used to produce the NUV geometric correction. The deuterium spectrum from a pre-launch test on 28 June 2012 was used to produce the FUV geometric distortion correction. The wavelength non-linearity component of this correction, however, is based on flare spectra observed on 11 October 2013, which show fluorescence-enhanced neutral atomic and molecular lines (Jaeggli, Judge, and Daw, 2018).

### Implementation

Level 1 data are dewarped during iris_prep. During this step, a static distortion map for each channel is used to resample the data from the original detector pixels using a cubic interpolation with the IDL interpolate function.

### Discussion

The positional error in dewarping should produce positional errors smaller than 0.1 pixels over the entire detector area, but systematic intensity errors are introduced by resampling, and they may become noticeable for high-precision observations.

In the standard data pipeline invoked by iris_prep, despiking is not performed prior to dewarping, primarily because automated despiking has the potential of removing real solar features. On the other hand, the resampling technique used to apply the distortion map broadens spikes caused by energetic particles, making them less discrete and more difficult to identify and remove from processed data. Nevertheless, spikes in spectra are typically still identifiable as such on closer inspection.

## Wavelength Calibration

**Figure 17:**
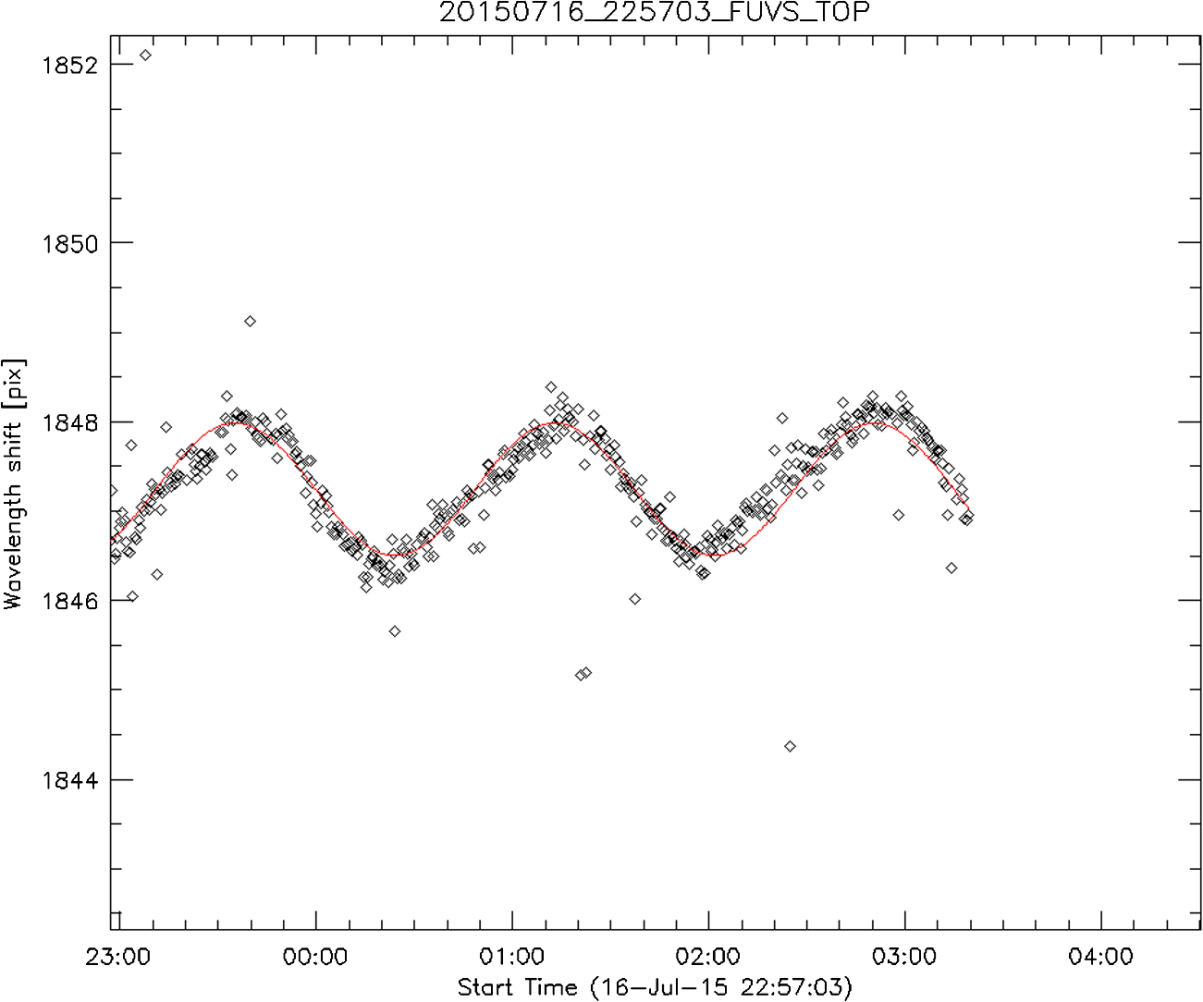
Example of wavelength measurements and best-fit calibration function.

The wavelength associated with a given pixel location on the IRIS spectrograph CCDs varies with time as a result of thermal effects. This is a source of velocity error if not properly calibrated. The goal of the calibration is to provide the data analysis software with accurate wavelength information through the header of the Level 2 FITS files. The IRIS pipeline software (*i.e.*, iris_prep) is currently using two different wavelength calibration methods, depending on the characteristics of the observations. The primary calibration method is a three-step process that runs automatically in the science data pipeline. This process is carried out separately for each observing sequence (OBS). In the first step of the process, the software measures the pixel location of a chosen spectral line in the spatially averaged spectrum of each SG frame of the given OBS. In the second step, it fits a sine function with a one-orbit period to all the line location measurements from the first step. The one-orbit period accommodates orbital Doppler velocity variations as well as orbit-induced thermal variations in the spectrograph alignment. The purpose of the fitting process is to reduce the noise inherent in the individual measurements from each frame. The third step applies the best-fit sine function to spectrally calibrate all frames of the given OBS. Figure 17 shows an example of wavelength measurements and the best-fit sine function used for the calibration.

**Figure 18:**
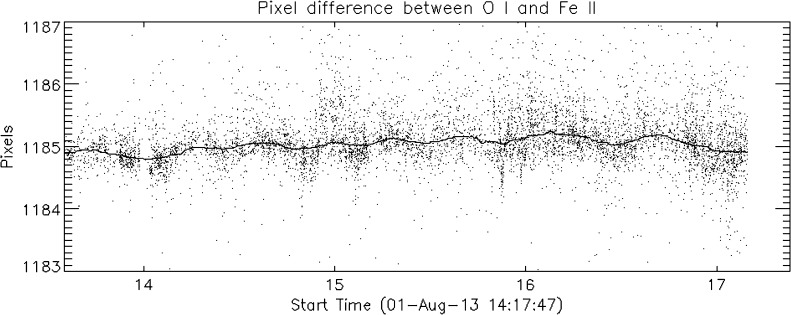
Offset measurements between the FUV-S and FUV-L detectors. The *solid line* is a 90-day running average. There is no systematic drift over the course of the mission.

The lines chosen for the primary wavelength calibration are a Ni i line at 2799.474 Å and an O i line at 1355.598 Å. In addition, an Fe ii line at 1392.817 Å is measured, but is not used for the calibration of the data because it is weak. Instead, the wavelength calibration assumes that the FUV-L (1389 – 1407 Å) CCD detector has a fixed wavelength offset from the FUV-S (1332 – 1358 Å) detector. However, we use Fe ii line measurements to track and verify this assumption. Figure 18 shows that there is no systematic drift between the two detectors. Most of the scatter is due to the uncertainty of the measurements. The 90-day running average (solid line) stays within a small fraction of a pixel, or about $\pm 0.5$ km s^−1^ over the first three years of the mission. The pipeline does not correct for these minute variations.

**Figure 19:**
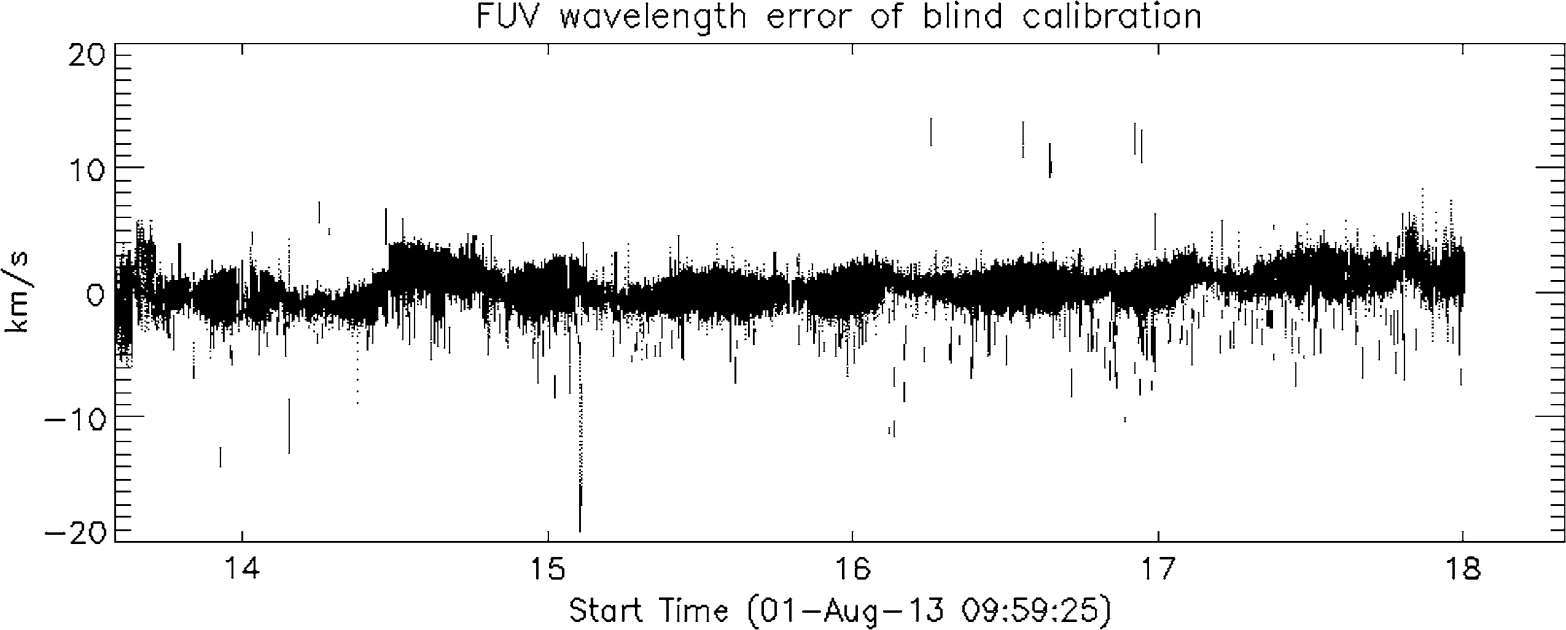
Difference between the primary and alternate wavelength calibration methods for the FUV SG over the first several years of the mission.

In about 90% of the NUV observations and 80% of the FUV observations, the primary wavelength calibration method is successful and leads to a velocity calibration accuracy of about 1 km s^−1^. For the remaining observations, an alternate, parameterized wavelength calibration model is used. It is based on instrument temperatures, pointing, roll orientation of IRIS at the time of the observations, and elapsed mission time. The alternate calibration method is always available, but it is typically less accurate and subject to slow secular drifts over the course of the mission. Figure 19 shows a scatterplot of the difference between the two wavelength calibration methods in the FUV. It reflects the calibration update that was the basis for the Level 2 data reprocessing effort in mid-2017. Earlier versions of the alternate calibration method showed a substantially larger error. Reprocessing in 2017 also fixed an issue where the wavelength calibration of the FUV-L range was redshifted by about 11 km s^−1^ relative to the (correctly calibrated) FUV-S range. It is therefore important to always use the latest version of Level 2 data. SolarSoft routines reading IRIS Level 2 data now alert the user if not the latest version of a data file is being ingested.

## Pointing, Fiducials, and Coalignment

### Slit Location and Alignment

Thermal variations in the instrument not only affect the wavelength calibration, but also the CCD pixel location of any spatial point on the slit. This motion of the slit image on the CCD affects i) the correction of any features on the slit during the flat-field process and ii) the accurate pointing of the CCD pixels relative to Sun center. To aid in the correction of these variations, the slit has two small gaps, or fiducial marks along its length, which can be detected in both the spectra and the slit-jaw images. The pipeline process that tracks the wavelength calibration also tracks the location of the slit in the SJI frames, as well as the location of the fiducial marks in the SJI and the SG frames. Similar to the wavelength correction, there is also an alternate parameterized model for this calibration. Both calibration methods work sufficiently well to obtain accurate pointing information. However, when this calibration was applied to the spatial component of the flat field (Section 4), the residual jitter introduced undesired noise to the flat-fielded data. Consequently, the calibration is not being used in the flat-field process.

### Absolute Pointing

Thermal variations on-orbit also affect the alignment between the guide telescope (GT) and the main telescope. Since the solar pointing is defined through the GT boresight, these variations affect the absolute pointing accuracy. Relative variations with an orbital period are corrected in the instrument in real time using the tip-tilt secondary mirror and an onboard wobble calibration table (Section 9.1). The absolute pointing, however, may still be off by some arcseconds. To reduce this error post facto, the calibration pipeline automatically correlates IRIS FUV SJI images with SDO/AIA 1700 Å images. The same pipeline process that determines the wavelength and slit position corrections also tracks the IRIS-AIA cross-calibration and creates a sinusoidal best-fit model for the absolute pointing of IRIS. The resulting pointing is tied to AIA, but the absolute accuracy is typically better than 1 arcsecond. This improved pointing information will be incorporated into the Level 2 data FITS header later in 2018, but has not yet been completed at the time of this writing.

## Absolute Throughput Calibration

The purpose of an absolute throughput calibration is to provide the data user with a means to convert observed intensities into absolute fluxes at the Sun. This knowledge may be an important factor in the interpretation of the observations in terms of physical processes. IRIS observations are not routinely converted into absolute units. Instead, the IRIS team provides the community with instrument-effective areas as a function of wavelength for each channel. As the instrument is aging, the effective areas have been changing, so the effective area values must be provided as a function of time. In addition to the effective area, the user must also know the gain of the CCD camera amplifiers in terms of data numbers (DN) per photon. The current best-estimate of the IRIS effective area for any given time is available in SolarSoft via the function iris_get_response.pro. The output of the routine also includes values of the CCD camera gain for each channel.

In the past, various methods have been used to radiometrically calibrate UV instruments. Suborbital rocket experiments are often calibrated in the laboratory shortly before launch, using calibrated standard detectors or sources (*e.g.* Kohl and Parkinson, 1976). On high-altitude balloon experiments where uncertain atmospheric attenuation is a concern, observed photospheric emissions may be compared with measurements from well-calibrated rocket payloads (*e.g.* Samain and Lemaire, 1985). The *Solar Ultraviolet Measurements of Emitted Radiation* (SUMER) instrument on the *Solar and Heliospheric Observatory* (SOHO) was primarily calibrated on the ground. However, the calibration was subsequently refined on-orbit i) by using solar line pairs at different wavelengths but with known intensity ratios and ii) by observing spectra of previously well-observed reference stars (Wilhelm *et al.*, 1997).

**Figure 20:**
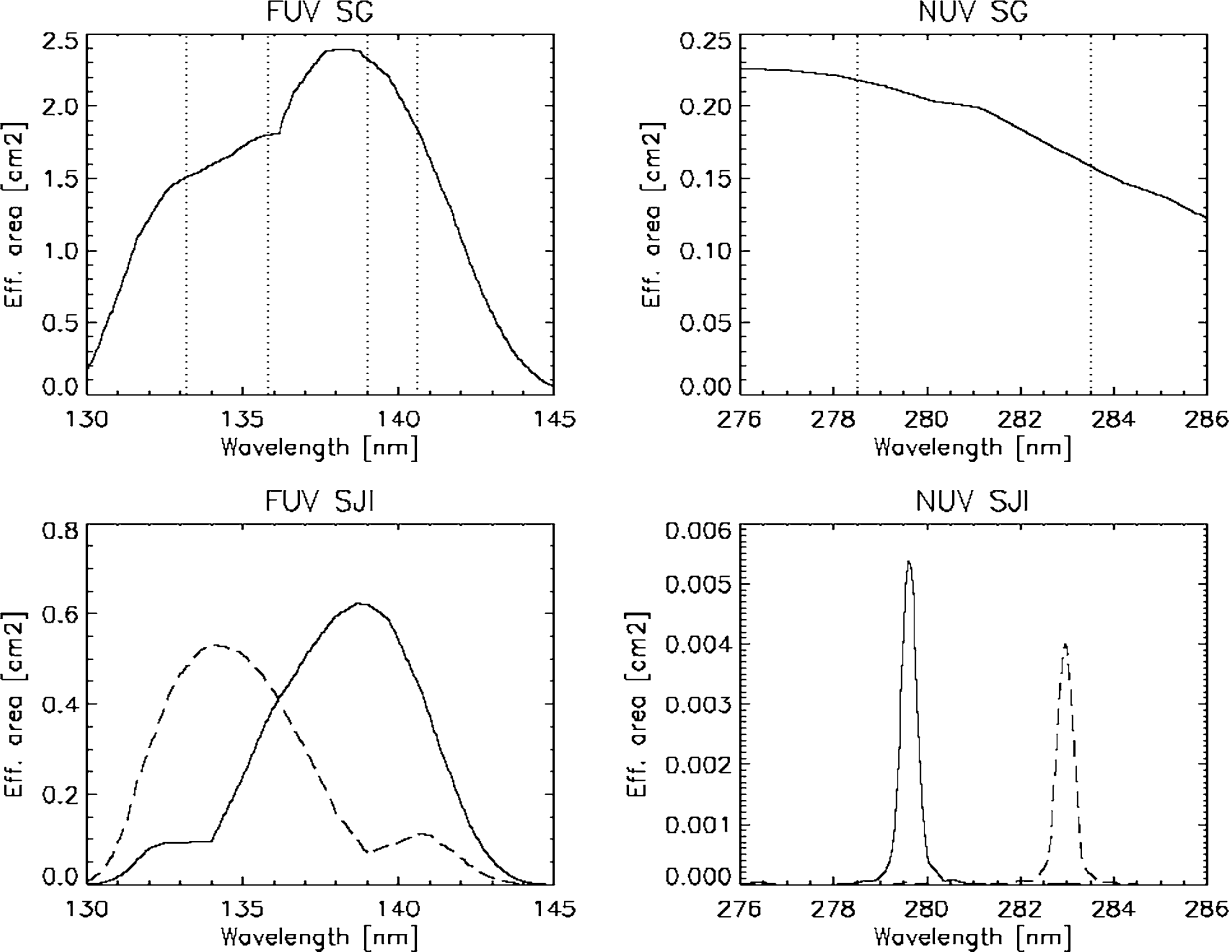
Estimates of the pre-launch effective area. The spectral windows of the spectrograph detector are indicated by *vertical dotted lines*. The *two curves in each of the bottom panels* indicate the response of each of the two FUV and NUV slit-jaw imager channels.

The IRIS spectral response and absolute throughput was initially derived from measured efficiency curves for each individual optical element of the IRIS instrument. These curves were then folded together and combined with the geometric telescope aperture to create effective area curves for each channel of the IRIS instrument (Figure 20).

Post launch, we are using two methods to measure and track the instrument throughput: i) by observing B-type stars and comparing the results with historic *International Ultraviolet Explorer* (IUE) or *Hubble Space Telescope* (HST) measurements, and ii) by carrying out a full-disk mosaic of the Sun and comparing the results with cotemporal *Solar Radiation and Climate Experiment* (SORCE)/*Solar Stellar Irradiance Comparison Experiment* (SOLSTICE) or *Thermosphere Ionosphere Mesosphere Energetics and Dynamics* (TIMED)/*Solar EUV Experiment* (SEE) measurements. B stars are bright enough only in the FUV, while SOLSTICE allows calibrations in both the FUV and NUV. We further found that the SOLSTICE cross-calibrations are more accurate and reliable than the stellar calibrations and chose them as the main method to calibrate IRIS. Nevertheless, the stellar calibrations are still useful as an independent verification method. The following subsections first outline the cross-calibration process with SOLSTICE and how the results are implemented in iris_get_response, the second subsection briefly discusses the stellar calibrations, and the third describes the CCD camera gain calibration.

### Cross-Calibration with SORCE/SOLSTICE

The SOLSTICE instrument on SORCE regularly measures the solar UV spectrum integrated over the solar disk with a spectral resolution of 1 Å (McClintock, Rottman, and Woods, 2005). The observed spectral range includes all IRIS spectral channels. During orbital night, SOLSTICE observes calibration stars to track and maintain the SOLSTICE absolute calibration. Unfortunately, the SORCE spacecraft experienced a battery failure just two days before IRIS first light, so SOLSTICE measurements simultaneous with IRIS were initially not available. The SORCE team later established a new observing mode that reinstated the SOLSTICE solar observations starting on 24 February 2014, although without the capability of measuring reference stars. The absolute accuracy of the SOLSTICE measurements after over a decade on orbit is estimated to be 5% (Snow *et al.*, 2005).

#### Spectral Cross-Calibration

**Figure 21:**
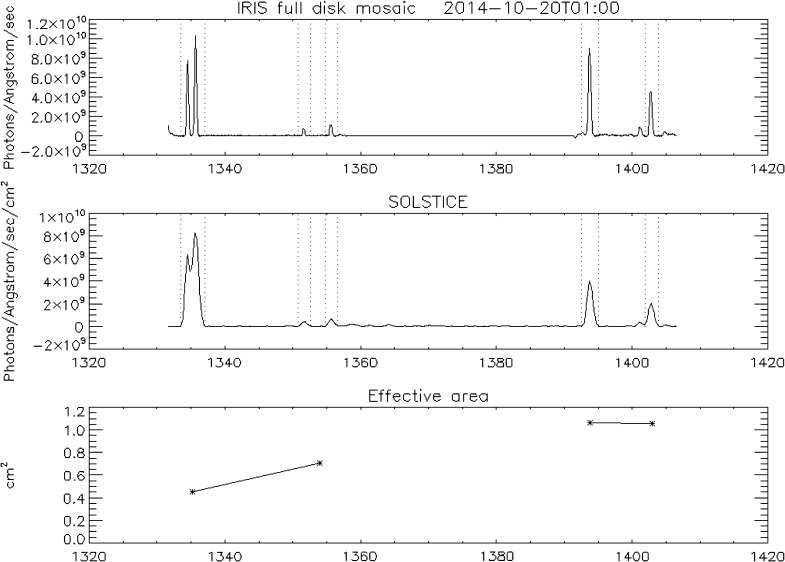
Full-Sun FUV spectra for 20 October 2014. *Top panel*: IRIS, *middle panel*: SOLSTICE, and *bottom panel*: derived IRIS effective area at four wavelengths (*star symbols*). *Vertical dotted lines in the top* and *middle panels* indicate the spectral regions used for the cross-calibration.

**Figure 22:**
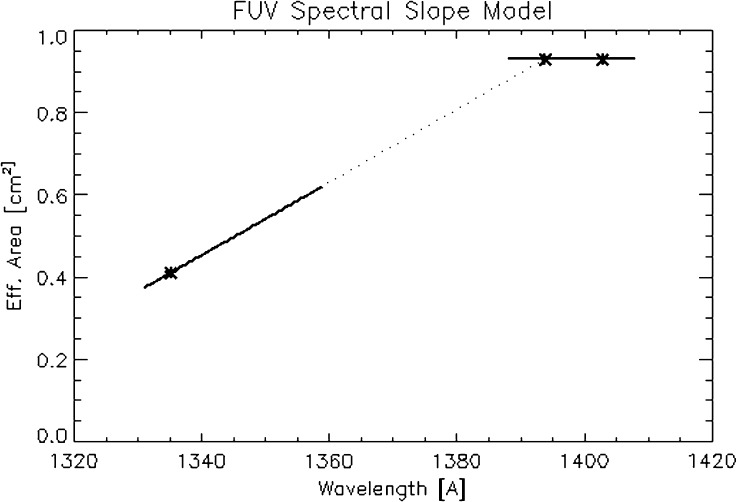
Model used for the calibration of the FUV SG (shown here with values for 1 March 2015). See text for details.

Figure 21 shows the disk-integrated solar FUV spectrum on 20 October 2014 observed by IRIS (top panel) and SOLSTICE (middle panel). The two spectra are not strictly simultaneous as the IRIS full-disk mosaic was acquired over a 14-hour time period. The IRIS spectrum is smoothed, while the SOLSTICE spectrum is shown at full resolution. SOLSTICE partly resolves the two C ii lines around 1350 Å and mostly separates the O iv line near 1401 Å from the Si iv line near 1403 Å. For cross-calibration purposes, we find that we can use the total of the two C ii lines, each of the two Si iv lines (at 1394 and 1403 Å), and the total of the Cl i, the O i, and the C i lines (between 1351 and 1356 Å). This provides us with four calibration wavelengths where we can determine the ratio of observed IRIS flux and observed SOLSTICE flux. The SOLSTICE flux has been calibrated in terms of photons Å^−1^ s^−1^ cm^−2^, so this ratio provides the IRIS effective area. It is indicated by stars in the bottom panel of Figure 21. Since there are two calibration wavelengths in each of the two IRIS FUV spectral windows, one could use a linear fit through each pair of data points to obtain the best-estimate effective area curves. However, we found that the measurements near 1354 Å show a substantial amount of scatter, probably because the lines used for the calibration are relatively weak. We also see some random measurement-to-measurement variability of the slope between 1394 and 1403 Å that is unlikely to be real. It is more likely that the true slope near 1400 Å does not change with time and is, in contrast to the pre-launch calibration, close to being flat. For the IRIS FUV calibrations, we therefore adopted the more robust model shown in Figure 22, in which: i) The spectral response of the long (1389 – 1407 Å) FUV range is flat and is determined by the weighted average response at the two Si iv lines. ii) The spectral response of the short (1332 – 1358 Å) FUV range is linear and the slope is determined by the C ii calibration at 1335 Å, and the Si iv calibration at 1394 Å.

**Figure 23:**
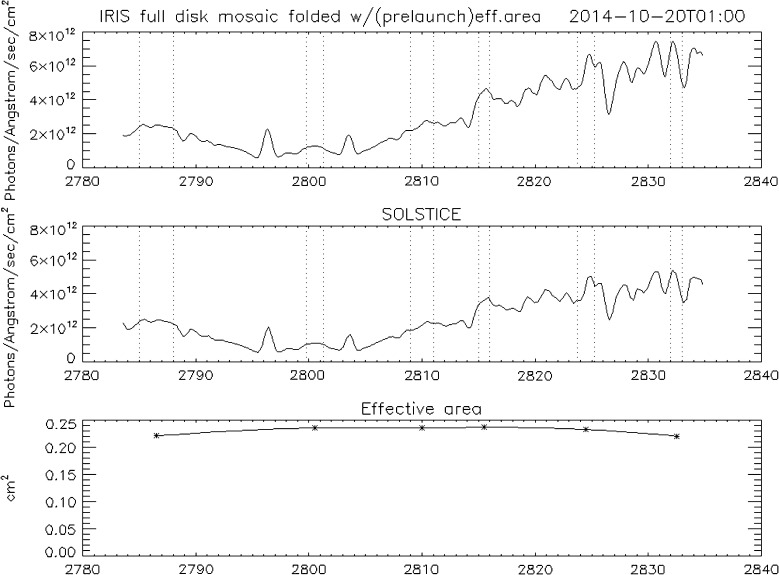
Full-Sun NUV spectra for 20 October 2014. *Top panel*: IRIS, *middle panel*: SOLSTICE, and *bottom panel*: derived IRIS effective area. *Vertical dotted lines in the top* and *middle panels* indicate the spectral regions used for the cross-calibration.

In the NUV, the solar spectrum shows substantial emission throughout most of the range of the IRIS spectrograph, and the cross-calibration with SOLSTICE is not limited to a few bright lines. Figure 23 shows the disk-integrated NUV spectrum from IRIS (top panel) and SOLSTICE (bottom panel) for 20 October 2014. The bottom panel indicates the derived IRIS effective area at six different wavelengths using stars. We found that the absolute effective area does vary with time, but the shape of the spectral response does not. For calibration purposes, the relative spectral response is derived from the average of the 2014 IRIS–SOLSTICE cross-calibrations, using a spline function to interpolate in wavelength between the six wavelength points shown in the bottom panel of Figure 23.

#### Spectrograph Trending

**Figure 24:**
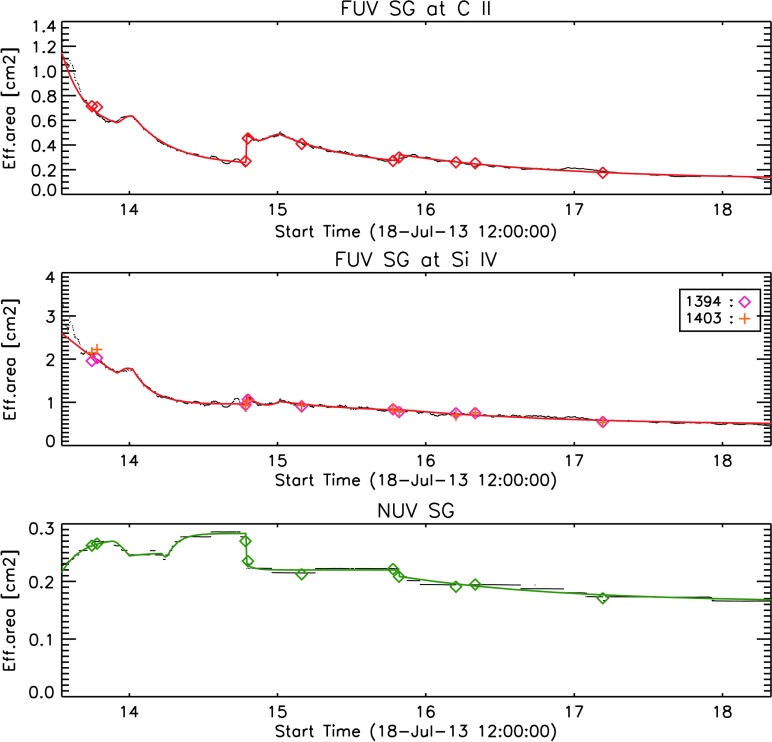
Trending of the IRIS SG response over the course of the mission. *Top panel*: FUV near 1335 Å, *middle panel*: FUV near 1400 Å, and *bottom panel*: NUV. *Colored diamonds* and *crosses* indicate absolute cross-calibrations with SOLSTICE, *black dots* are scaled running averages of IRIS quiet-Sun observations, and *colored solid lines* are the final parametric fits. See text for details.

In the previous paragraphs, we described our simplified spectral models for the FUV and NUV spectrograph calibrations. These models reduce the time-dependent variables to only two in the FUV and one in the NUV, or essentially the average IRIS response in the three spectral windows around 1335 Å, 1400 Å, and 2800 Å. The IRIS response for these wavelength windows over time is shown in the three panels of Figure 24. The colored symbols in each panel indicate the results of the IRIS–SOLSTICE cross-calibrations.

Full-disk mosaics are very time consuming, and IRIS–SOLSTICE cross-calibrations are performed only a few times *per* year. To monitor the medium-term variability of its response, IRIS takes daily standardized observations of a quiet-Sun region near disk center that provide average quiet-Sun intensity values at the three calibration wavelengths. Because of solar variability, these values show substantial scatter, which can be smoothed by a running monthly average. These smoothed intensities are a useful means to track the IRIS response between SOLSTICE cross-calibrations, but in the long run, they do not accurately track the cross-calibration results, in particular in the FUV. This is presumably due to the fact that the typical quiet-Sun intensity in the UV depends on the solar cycle. However, we can capture both medium- and long-term trends by multiplying the smoothed quiet-Sun intensities with a function that varies only on solar cycle timescales. The function is chosen from a best fit to the SOLSTICE cross-calibrations. The resulting smoothed and adjusted quiet-Sun intensities are shown in all panels of Figure 24 as black dotted lines.

In a final step, we fit parametric functions to these intensities (colored solid lines in Figure 24) that provide the time variability for our spectral response model in iris_get_response.pro.

It is worth discussing a few features of the temporal evolutions in Figure 24. i) The FUV SG response declines by about a factor of two over the first nine months of the mission, but then starts leveling off. This initial decline may be related to residual outgassing of the observatory condensing, and possibly polymerizing on the optics. The FUV response changes in the very early portion of the mission are not very well understood as the first absolute calibration did not occur until 2.5 months after first light. In general, the post-launch effective area near 1335 Å appears to be somewhat lower than the pre-launch effective area value of 1.5 cm^2^, while near 1400 Å, the post-launch area appears to be slightly larger than the pre-launch value of 2.1 cm^2^. ii) Both FUV curves show a slight response gain toward the end of each year, around the time of the beginning of the eclipse season (*i.e.* the time of the year when the Sun is eclipsed by the Earth for several minutes every orbit). iii) The 1335 Å curve shows a sudden response increase in the Fall of 2014. This change was caused by the first IRIS detector bake-out. It appears that a good portion of the sensitivity decrease at that time was caused by a thin layer of contamination on the CCDs. Later bake-outs did not show nearly as much improvement. The material condensed on the CCD affected 1335 Å much more than 1400 Å, which is not surprising as molecular contaminants absorb more strongly at shorter wavelengths. iv) The NUV response does not show a long-term response decline. On shorter time scales it shows a nearly opposite behavior to the FUV response, with a sensitivity increase/decrease at times when the FUV shows the strongest decrease/increase. This phenomenon is not fully understood, but we hypothesize that it may be caused by a thin contamination layer on the CCD acting as an anti-reflection (AR) coating. When the layer thickness grows, as was the case early in the mission, the AR effect increases, while during the bake-out, the removal of the contamination layer from the CCD caused the AR effect to disappear. After three years on orbit, the NUV sensitivity appears to have returned to nearly the same value as at launch.

#### Slit-Jaw Imager

**Figure 25:**
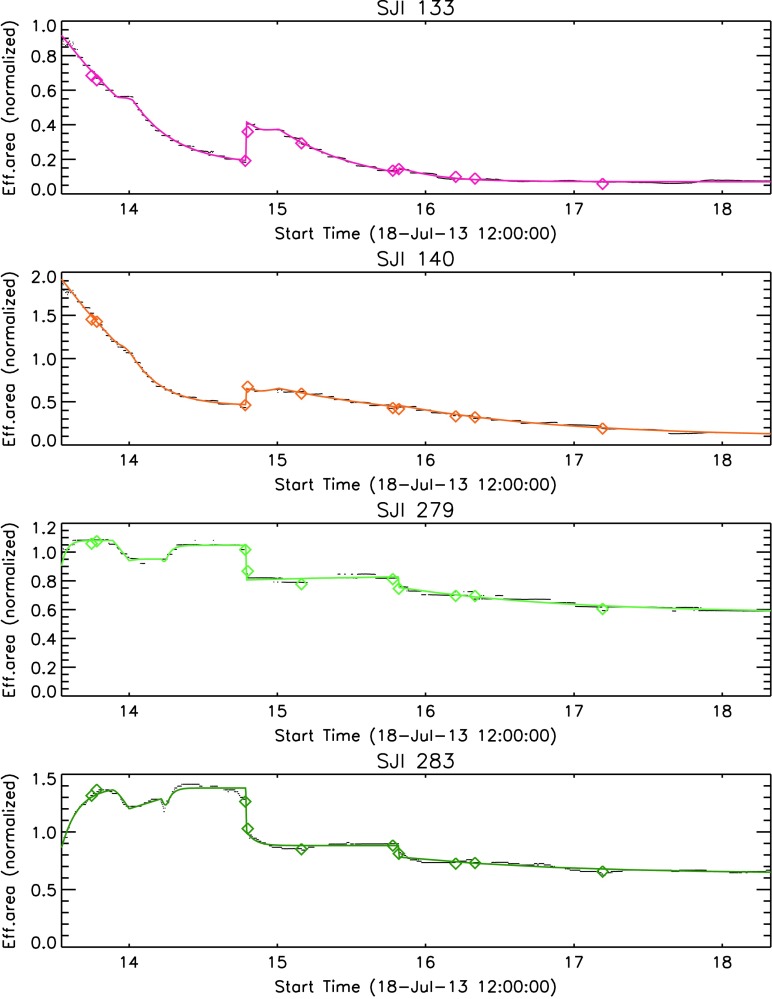
Trending of the response of the four IRIS SJI channels over the course of the mission. *Diamonds* indicate cross-calibrations with SOLSTICE, *black dots* are scaled running averages of IRIS quiet-Sun observations, and *colored solid lines* are the final parametric fits. See text for details.

Post-launch spectral calibration of the slit-jaw imager (SJI) is more difficult and more prone to error than the SG calibration. A component-wise calibration of the spectral response was carried out pre-launch, but it is likely that the actual (post-launch) response is noticeably different and/or may have shifted since the pre-launch measurements. This uncertainty predominantly affects the relative contributions of the C ii *versus* the Si iv lines to the images in each of the two FUV channels. We attempted to carry out a best estimate of the relative spectral response changes in the FUV SJI by assuming that they are similar to the changes in the FUV SG. We implemented this by calculating the ratio of actual *versus* pre-launch response for the SG at 1335 and 1400 Å, and then applied a linear correction based on those two ratios to the SJI pre-launch response curves. However, it is not clear that the SJI changes are the same as the SG changes. In fact, the SJI has a transmission filter that may age differently than the reflective surfaces used in the SG. The latter were found to be quite stable in tests, while the aging of the FUV transmission filter has not been thoroughly studied. Nevertheless, we use the SJI pre-launch transmission curves, corrected by the measured SG changes (as discussed above), as the basis of the effective area calibration. To obtain an absolute calibration value, these effective area curves were then scaled as a whole so that the integrated SOLSTICE spectrum folded with these adjusted SJI curves matches the total measured photon fluxes from the IRIS full-disk observations. The results are shown using color symbols in Figure 25. Medium-term trending was derived from the daily IRIS quiet-Sun observations in the same way as the SG trending, and is shown as black dotted lines in Figure 25. The colored solid lines show the functional fit that is used for the response calibration in iris_get_response.pro.

The most noteworthy findings from Figure 25 are as follows: i) The response change in the FUV SJI is more pronounced than in the FUV SG, with a decrease of about a factor of three over the first nine months of the mission. As in the FUV SG, the decline starts leveling off afterwards. ii) All channels show a pronounced change at the first detector bake-out. iii) The SJI 2832 Å (Mg ii wing) channel appears to vary more than the SJI 2796 Å (Mg ii line center) channel. This latter result is slightly puzzling. More analysis may be required to confirm this behavior. It is perceivable that the AR coating effect of a contamination layer on the CCD is more effective at the longer wavelength.

### Stellar Calibration

IRIS can observe objects up to about 5 arcmin above the solar limb without its *Guide Telescope* losing fine-pointing control. Over the course of a year, about a dozen sufficiently bright B stars pass the Sun within that range and provide an alternate throughput calibration opportunity. In addition to their UV brightness, the target stars have been selected for minimum variability and the availability of good spectra from at least IUE and preferably also from HST. We routinely observe HD19374, HD91316, HD142096, HD144470, and HD210424 with the FUV SJI, although we have observed a few others early in the mission. Only HD91316 ($\rho \ \mathrm{Leo}$) and HD144470 were bright enough for IRIS to obtain spectra with the FUV spectrograph and only during the first year of the mission. None of these stars are bright enough for NUV observations.

Our observing strategy (as suggested by John Raymond) is to place the IRIS slit a few arcseconds west of the star, let the star transit the slit, and then repeat the process. Up to about 20 such slit transits are possible over the course of several hours. At each transit we acquire a series of exposures in the FUV SG and the two FUV SJI channels. The images provide the calibration data for the SJI and allow us to locate the proper spectrograph exposure and location when the star passed the slit. The slit transit time is about 10 s. In August and November 2013, we observed 17 spectra of HD91316 and 3 spectra of HD144470, respectively.

To derive an IRIS effective area, we first predict the total count rate for the star in the IRIS spectra by folding the calibrated IUE spectrum of the star with the pre-launch effective area of IRIS. We then divide the total observed counts in the IRIS spectrum by the slit transit time and compare this observed rate to the predicted rate. The IRIS spectra are too noisy to obtain the spectral shape of the FUV SG response, but sufficient to obtain effective areas separately for the FUV-L (1389 – 1407 Å) and FUV-S (1332 – 1358 Å) spectral ranges. The observed count rates were very similar to the predicted ones for the FUV-L range, and slightly lower for the FUV-S range. The derived effective areas were 2.2 cm^2^ for the FUV-L and 1.2 cm^2^ for the FUV-S. This compares very well with the effective areas derived from the SOLSTICE calibration of 2.3 and 1.1 $\mathrm{cm^{2}}$, respectively. The results from HD144470 were similar but noisier and therefore less accurate.

Stellar observations with the slit-jaw imager have been possible throughout the mission thus far. These stellar calibrations qualitatively appear to track the results from the SOLSTICE calibration. However, the implied loss of sensitivity over the course of the mission is roughly a factor of two higher than the SOLSTICE calibration indicates. The discrepancy is not well understood, but we have much more confidence in the accuracy of the SOLSTICE cross-calibration. First, the SOLSTICE results are not hampered by low count rates and associated noise as the stellar results are. Second, the SOLSTICE calibration is carried out with the Sun and the same spectral lines that IRIS primarily observes. In contrast, the stellar calibration uses the spectra of B stars, which have a strong FUV continuum. This would not be an issue if we had accurate knowledge of the SJI filter response as a function of wavelength. Unfortunately, this is not the case. The spectral response of the SJI channels cannot be measured on orbit and has likely changed from pre-launch as a result of contaminants and the aging of coatings. As we are using the pre-launch response curves to predict the SJI count rates for the B star, we are introducing a considerable source of error, especially several years into the mission.

For more details and the result of the stellar calibration, we refer to IRIS Technical Note (ITN) 24 at http://iris.lmsal.com/documents.html.

### Gain Calibration

The calibration of solar observations in terms of absolute units requires knowledge of the instrument effective area, the instrument spatial and spectral plate scales, and finally the CCD camera gain. Most commonly, we use the inverse camera gain, $j$, for converting the number of detected photons, $P$, into data units, $S$:

6 $$ S = P /j. $$

The (inverse) camera gain is typically measured via a photon transfer curve (PTC). The PTC takes advantage of the fact that the noise in a CCD camera is dominated by the shot noise at moderately high signal levels. This leads to the following relationship between shot-noise-induced signal variance, $\sigma ^{2}_{S}$, and signal level:

7 $$ \sigma ^{2}_{S} = S/j. $$

**Figure 26:**
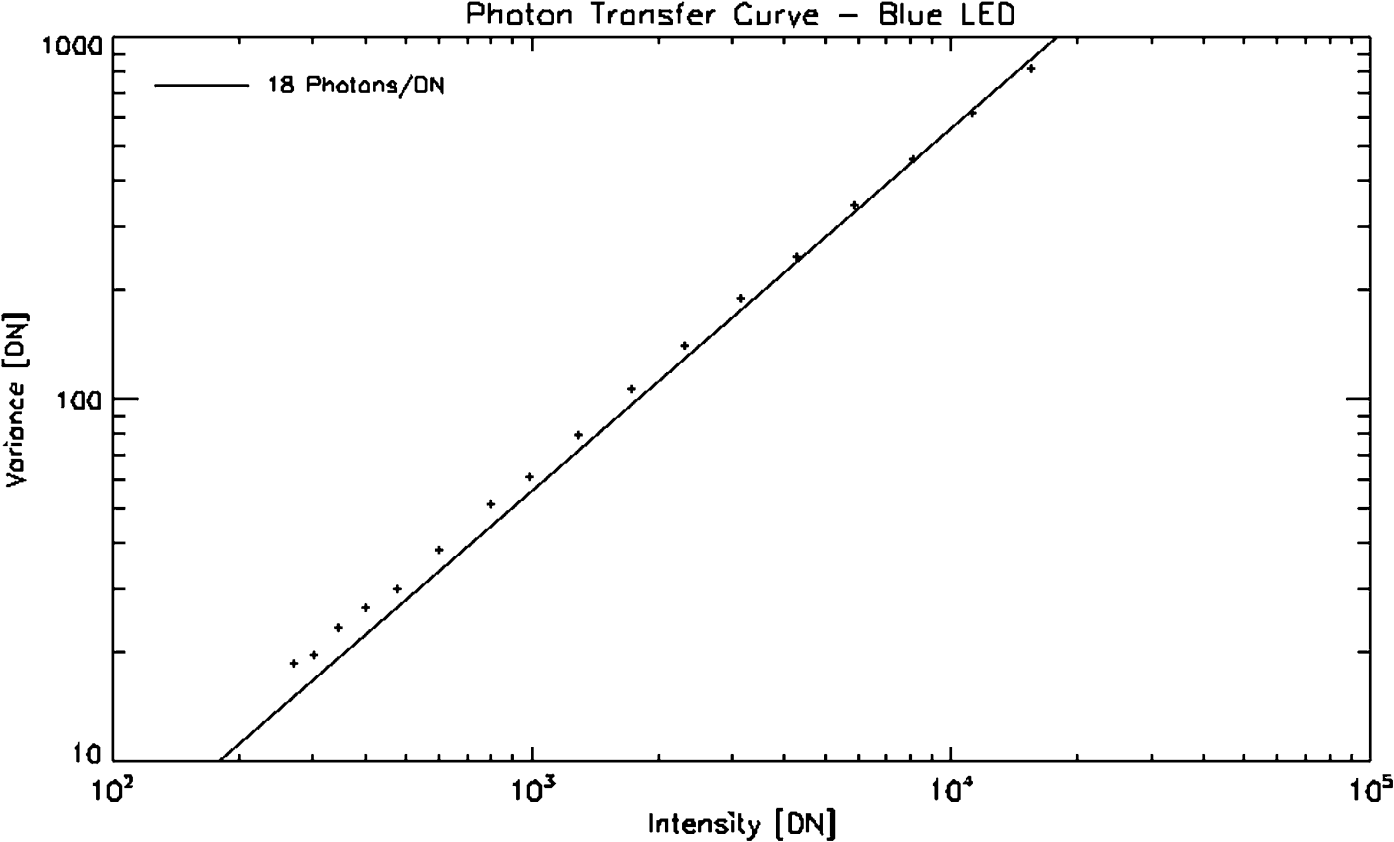
Photon transfer curve for the SJI CCD measure with a blue LED.

A PTC can be generated by imaging a constant light source with multiple images at each of a range of exposure times. The average signal variance is plotted against signal for each signal level. The PTC curve will deviate from the idealized curve at short exposures because of the camera read noise and at long exposures because of camera non-linearities. Figure 26 shows a PTC curve for the SJI CCD illuminated by the onboard blue LED shortly before IRIS first light. The (inverse) CCD camera gain was close to 18 photons DN^−1^. We have repeated this measurement at various times over the mission and found no change. The NUV SG CCD uses the same camera gain, while the FUV SG camera uses an electronic gain that is three times higher for increased sensitivity at low count rates.

**Figure 27:**
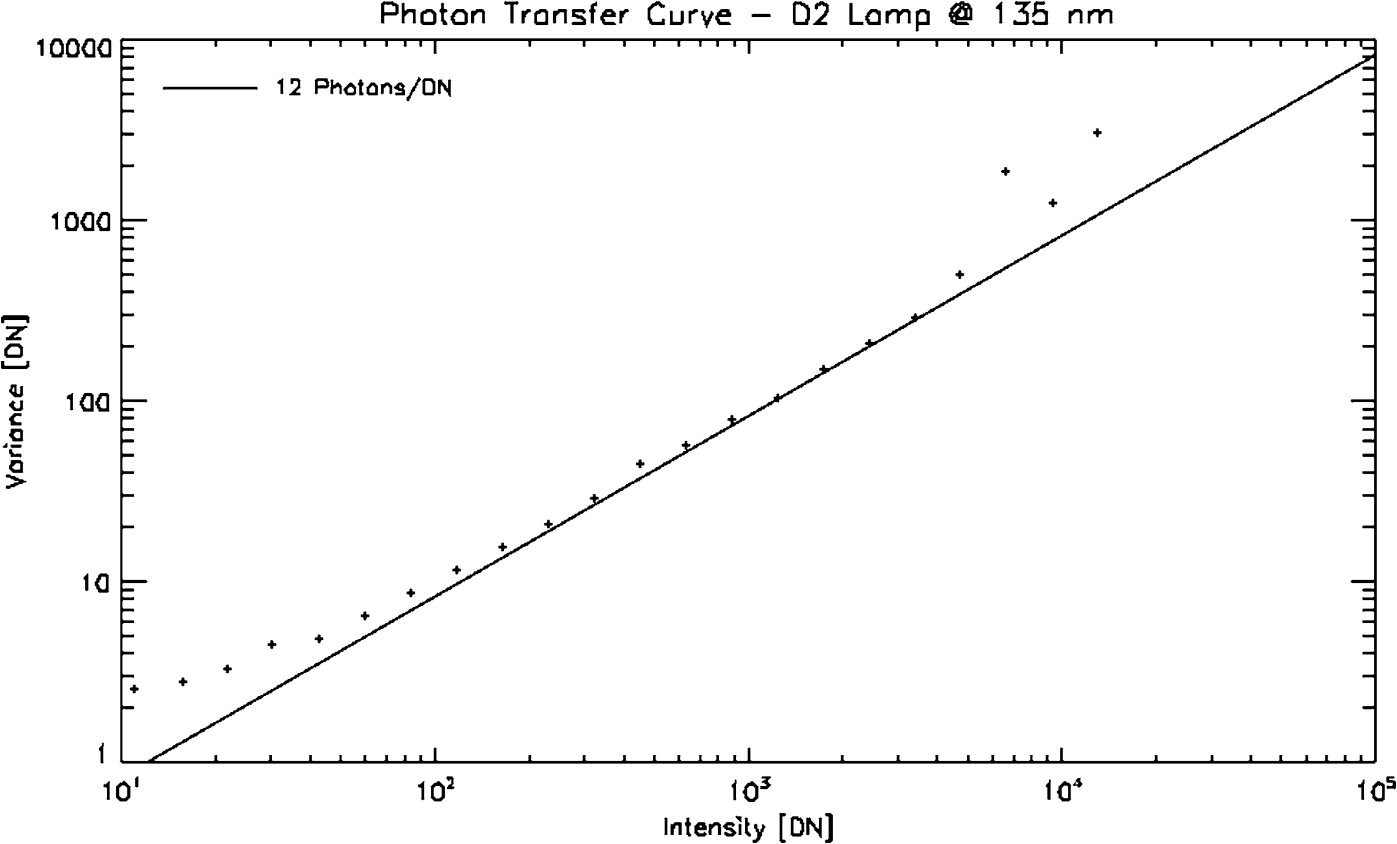
Photon transfer curve for the SJI CCD measured with a deuterium lamp at 1350 Å.

The CCD camera gain is essentially constant over a range of photon energies from the infrared to the near UV. At higher photon energies, however, a single photon can create more than one electron-hole pair, which causes the gain to increase (or the inverse gain to decrease). Figure 27 shows a PTC curve for the SJI 1330 Å channel illuminated by a deuterium lamp during ground testing. The resulting FUV (inverse) gain is 12 photons DN^−1^, *i.e.* about a factor 1.5 different from blue light. We have adopted this factor for all of our FUV calibrations.

On closer look, however, the factor 1.5 gain difference is less than expected, since the energy of the FUV photons is sufficient to create at least two electron-hole pairs. We suspect that charge spreading in the CCD substrate may occasionally allow one of the two simultaneously created photo-electrons to migrate into a neighboring pixel. This effect would artificially reduce the apparent shot noise and the resulting gain factor in the PTC measurement. We have recently analyzed PTC measurements with larger pixels to reduce the effect of charge spreading. The results suggest that the gain factor at 1350 Å may indeed be as high as 2. We have not measured a PTC at 2800 Å, but the NUV gain is expected to be very similar to the gain in the blue.

Table 1 summarizes the adopted gains for the various IRIS channels. As the table shows, we are currently still using a factor of 1.5 for the gain in the FUV, not the potentially more accurate value of 2. It is important to note that an error in this factor does not affect the accuracy of the absolute cross-calibration results with SOLSTICE. Owing to the nature of the cross-calibration process, an error in the CCD camera gain is compensated for in the effective area results of the calibration. A gain factor of 2 instead of 1.5, for example, would result in 25% lower FUV effective area numbers. The gain factor does, however, affect estimates of the Poisson noise.

**Table 1:** (Inverse) CCD camera gains for all IRIS channels in photons DN^−1^.

	Spectrograph	Slit-jaw imager
NUV	18	18
FUV	4	12

## Other calibrations

### Wobble Calibration

The thermal conditions at IRIS vary over the course of an orbit as a result of the satellite orientation with respect to terrestrial albedo. These thermal variations induce bending in the mounting of the guide telescope, which in turn introduces a pointing wobble that is a function of the orbital position. The wobble varies annually, but is relatively stable on a weekly timescale. Over an orbit, typical magnitudes of the wobble are 2 – 4 arcsec in $x$ and 1 – 2 arcsec in $y$. The thermal conditions of the satellite vary more dramatically during the eclipse season, resulting in larger changes in the wobble over an orbital period (up to 8 arcsec in $x$ and 3 arcsec in $y$) as well as more significant changes to the magnitude of the wobble on a weekly to monthly timescale. The roll angle of the satellite also affects the wobble since the orientation of the guide telescope is altered when IRIS is rolled. As described below, the wobble under rolled conditions is phase shifted from that when not rolled.

In contrast to the calibrations discussed earlier, the wobble calibration is not applied to the data post facto. Instead, a periodic, orbital-phase-dependent pointing correction is applied to the telescope secondary mirror in real time.

The first stage of the calibration procedure to correct for the pointing wobble is to quantify its effect. We measure the on-orbit wobble by collecting SJI 2832 Å channel images over two successive orbits with 10 s exposure time and 20 s cadence. We quantify the wobble in the $x$- and $y$-directions independently by performing this observing routine at both the east limb and the north pole, such that the limb of the solar disk is in the field of view, and running a cross-correlation algorithm on the data. These results are used to create an orbital wobble table (OWT) that consists of corrections to the piezoelectric transducers (PZTs) of the secondary mirror to compensate for the wobble.

**Figure 28:**
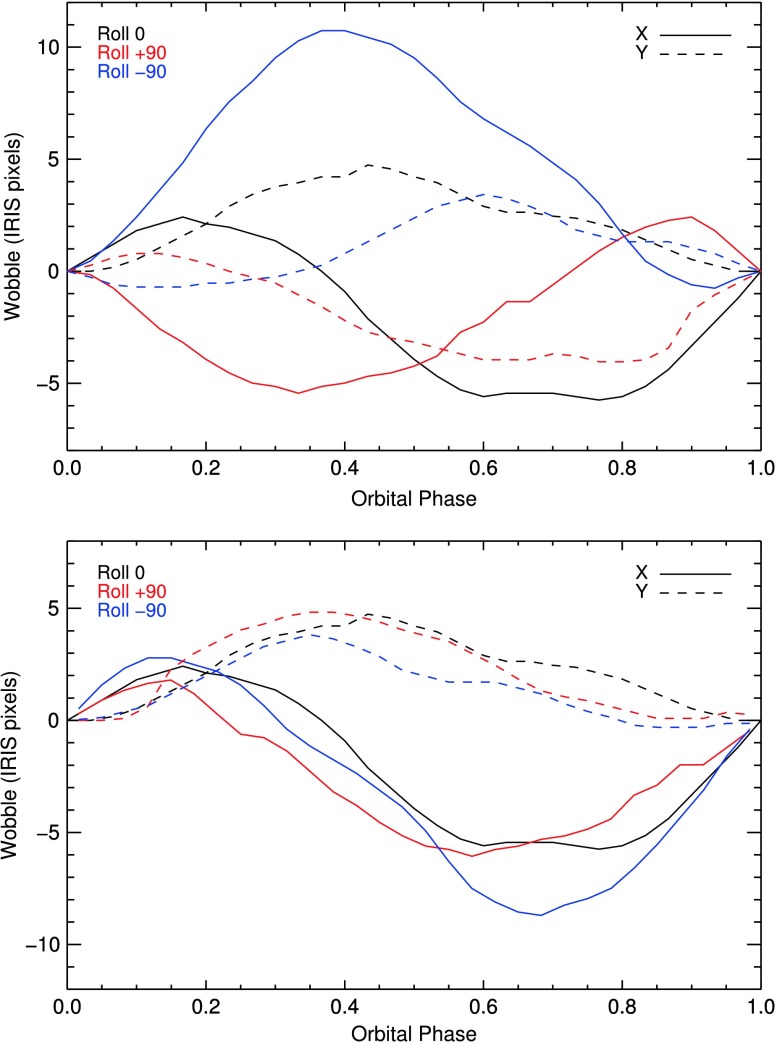
Orbital wobble correction. *Top panel*: IRIS orbital wobble in units of IRIS 0.16 arcsec pixels, for the $x$- (*solid lines*) and $y$- (*dashed lines*) directions, and three roll angle values: 0 (*black*), $+90$ (*red*), and −90 (*dark blue*). *Bottom panel*: Same curves as in the top panel, but with a phase shift of the wobble curves for $+90$ and −90 of $+0.25$ and −0.25, respectively. The shifted curves are very similar to the wobble curves for $0^{\circ }$ roll angle. The orbital phase is zero at the time when IRIS passes through its ascending node.

The above analysis has been performed at roll angles of $0^{\circ }$, $\pm 45^{\circ }$, and $\pm 90^{\circ }$. Figure 28 shows the results of these analyses for October 2015. The magnitude of the wobble shown here is typical of that during non-eclipsed observations. When in eclipse season, the wobble is on the order of 10 arcsec. The magnitude of the wobble is similar across different roll angles, but shifted in phase. For a roll angle of $\alpha $, the wobble can be approximated by the wobble for $0^{\circ }$ roll angle, shifted in phase by $\alpha /360^{\circ }$. This is illustrated in the bottom panel of Figure 28. This property allows us to correct for the orbital wobble at an arbitrary roll angle without overburdening the IRIS science observations with calibration routines. When rolled to angle $\alpha $, we correct for the wobble by applying the $0^{\circ }$ OWT shifted in phase by $\alpha /360^{\circ }$. The exception to this is for $\alpha =\pm 90^{\circ }$. Because $\pm 90^{\circ }$ rolls are used more frequently than other angles and the phasing formula decreases in accuracy with the degree of roll, we perform the calibration routine separately for $\pm 90^{\circ }$ and generate separate OWTs for these angles.

**Figure 29:**
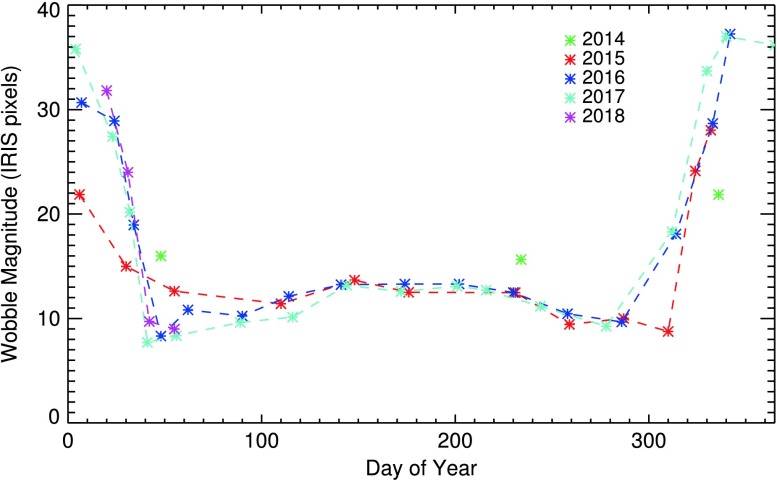
Variation of the wobble over the mission at a roll angle of $0^{\circ }$. Measurements for each year are shown in different colors. The wobble magnitude is defined as the absolute value (in Euclidean space) of the drift within an orbit. The *asterisks* indicate where orbital calibration data were taken with *dashed lines* to guide the eye on the annual variation. There were only three calibrations in 2014, so we have not connected these data points with lines.

Figure 29 shows how the magnitude of the wobble has varied over the mission. Shown here is the magnitude of the wobble over an orbit, *i.e.* the $\sqrt{x^{2}+y^{2}}$ drift within an orbital period. Wobble calibration data are sparse for 2014, but for the last three years, we see that the annual variation in the wobble has been consistent from year to year. The effect of eclipse season (November to February) on the magnitude of the wobble is also evident. Given the timescale of the wobble variation, calibration observations for roll angles of $0^{\circ }$ and $\pm 90^{\circ }$ are normally performed on a monthly basis. In addition, the wobble correction is checked weekly and more frequent calibrations are performed as needed, notably when entering and exiting the eclipse season.

### Polarization

Although IRIS is not a spectropolarimeter, it was expected that the FUV and NUV gratings may both act as partial linear polarizers. There are phenomena on the Sun (*i.e.* strong magnetic fields in active regions, scattering, and atomic level polarization) that induce polarization in spectral lines and continua. The Mg ii lines in the NUV are expected to have a fairly strong linear polarization signal (as much as 10%) based on recent spectral synthesis (Belluzzi and Trujillo Bueno, 2012). To assess the potential amplitude of polarization-induced intensity changes in the Mg ii line measured with IRIS, we characterized the efficiency with which the NUV grating acts as a polarizer.

The linear polarization response of the NUV spectrograph was measured during optical integration and testing prior to launch. A 2796.74 Å laser was used to provide light 100% polarized in one direction. A half-waveplate retarder optimized for 2660 Å was used to modulate the linear polarization direction from the laser, and was placed in the optical setup of the stimulus telescope (StimTel) that illuminated the IRIS instrument for ground testing. The StimTel was aligned with the IRIS telescope, and the laser spot was placed in the middle of the slit as verified by the NUV slit-jaw imager. The focus was adjusted to provide a larger and less intense laser spot so that the laser in the spectrograph images would not saturate. Four different series of measurements were taken. For each measurement, the waveplate was adjusted from $0^{\circ }-90^{\circ }$ in increments of $5^{\circ }$. The waveplate was mounted in a simple rotation mount, and adjustments were made by hand. Because the placement of the waveplate made the scale difficult to see, the adjustments may be imprecise at the $2^{\circ }$ level.

**Figure 30:**
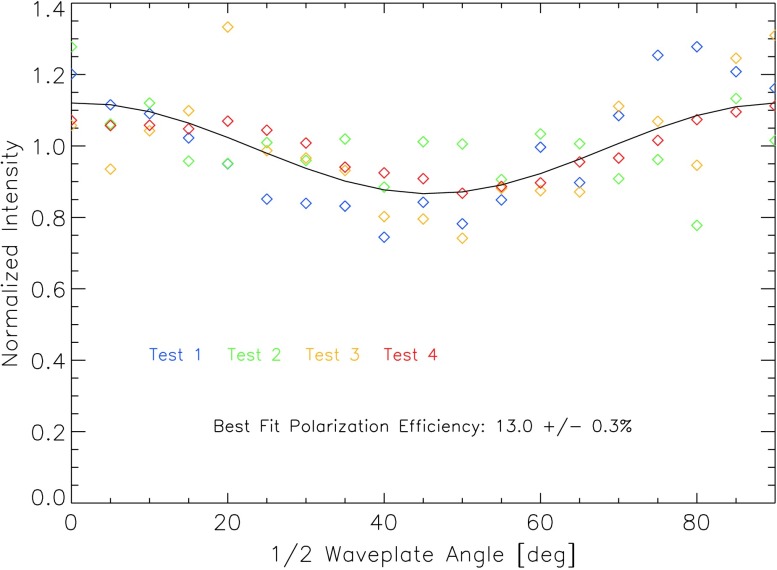
Intensity of the laser spectrum as a function of the half-waveplate orientation for the four measurement series (*colored points*). The fit to the data is shown by the *solid black line*.

The intensity of the laser was totaled, totals from an adjoining region of the same size with no signal were subtracted to account for the background signal. Figure 30 shows the results from the four series in different colors. It is immediately apparent that there is a great deal of noise in the intensity measurement. This is due to drift of the laser across the slit. During the first three sets of measurements (yellow, green, and blue), the optical table was floated, but during the final set of measurements (red), the table was settled on its supports. In the spectrograph images it appears that the laser spot was drifting across the slit, leading to a change in the illumination pattern and intensity level.

We adopted the manufacturer’s value for the retardance at the laser wavelength, and fit the observed intensity for the grating polarization efficiency, $e$, and an additional angle term to account for an offset error between the laser and the waveplate. The fit is shown by the black line in Figure 30. The resulting polarization efficiency for the grating for this fit is 13% perpendicular to the groove direction of the grating. When we combine this polarization sensitivity with a potential linear polarization signal of 10%, we find that the resulting polarization induced intensity change would be only about 1.3%.

### Miscellaneous

**Figure 31:**
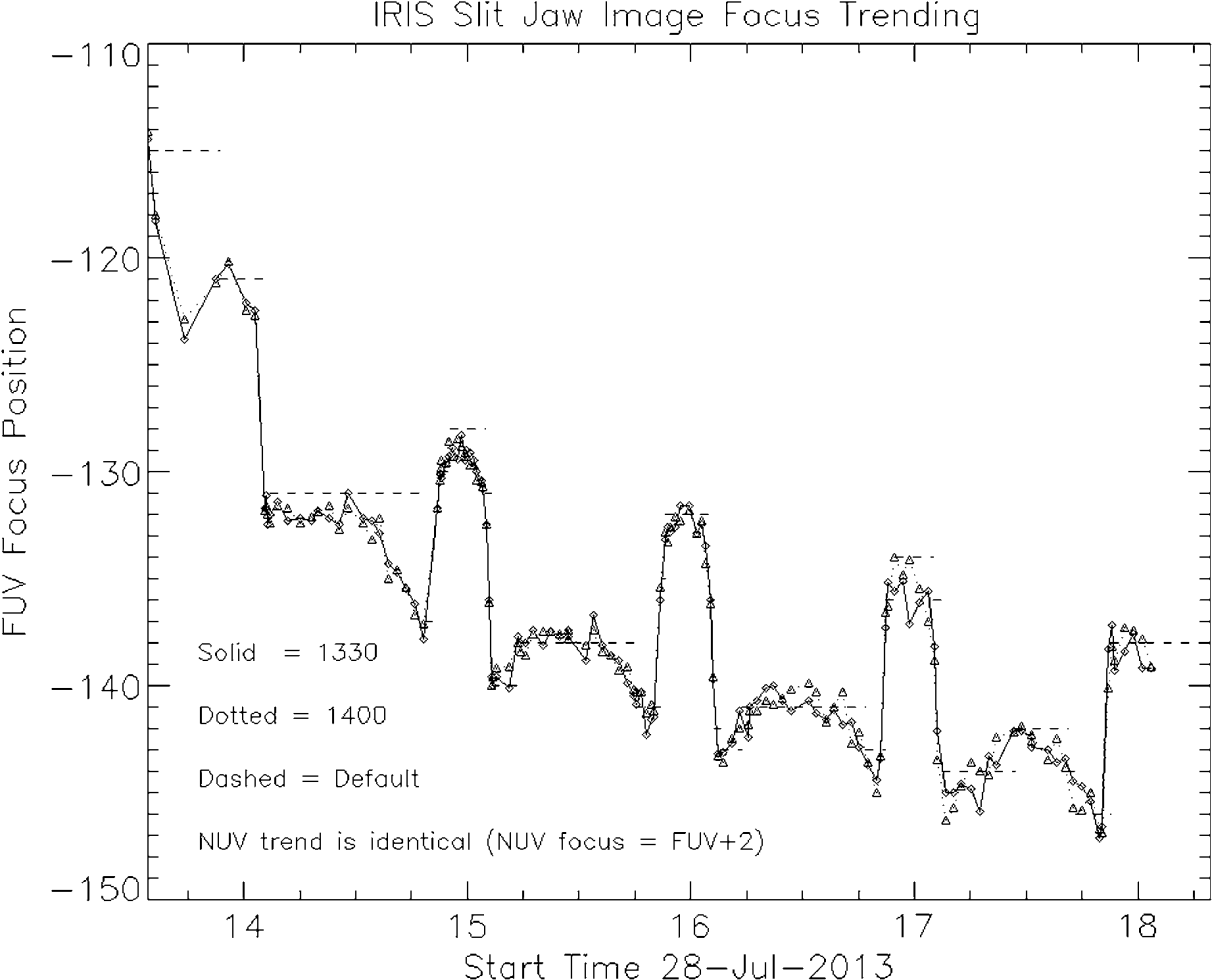
Evolution of the setting for best focus (in focus motor steps) over the mission. The changes are primarily due to variations of the thermal environment. The annually recurring jumps are caused by the eclipse seasons.

In addition to the calibrations discussed above, the IRIS team performs periodic calibrations of onboard system settings, such as the PZT actuator gains of the image stabilization and spectrograph raster scan system, and the focus setting of the IRIS telescope. These systems are very stable on short terms, but show slow drifts due to seasonal variations of the instrument thermal environment. As an example, Figure 31 shows the evolution of the setting for best focus of the IRIS telescope.

Other calibrations or corrections applicable to the IRIS data include the characterization of the instrument point spread function (PSF) and the dust particle removal process that may be applied to the raw slit-jaw images.

The PSF of the IRIS spectrograph was first characterized pre-launch in the laboratory and recently in-flight using the 9 May 2016 Mercury transit. The results are documented in Courrier *et al.* (2018).

Dust particles that migrated onto the SJI CCD pre-launch result in speckle-like dark features in the SJI images. They can be removed on the data-user end by calling an IDL routine iris_dustbuster.pro, which fills the dust pixels with good-pixel values of the same location on the Sun from neighboring frames adjacent in time. This thus works best on coarse or many-step dense/sparse rasters, where good-pixel data are readily available. For sit-and-stares or narrow rasters, the routine still works to some extent, but fills the dust specks with a spatially blurred interpolation of the surrounding area. Dust removal is not routinely applied in the science data pipeline, but is applied to the quick-look imagery on the IRIS website. Note that the current dust-buster does not always work perfectly, because it uses a set of dust masks at fixed positions and does not take into account thermal drifts of the fiducials. In practice, the actual image data are placed so that the fiducials always remain at the same locations in the FITS files. At times when thermal flexing of the instrument causes the fiducials and thus the images to drift, this correction could fail and miss the dust by a few pixels. This shortcoming could be improved by allowing the dust masks to move with time to compensate for the thermal drifts.

For various calibration-related subjects not covered or only briefly mentioned in this article, we refer to the latest technical notes at http://iris.lmsal.com/documents.html, which are periodically updated.

## Data Processing Pipeline

**Figure 32:**
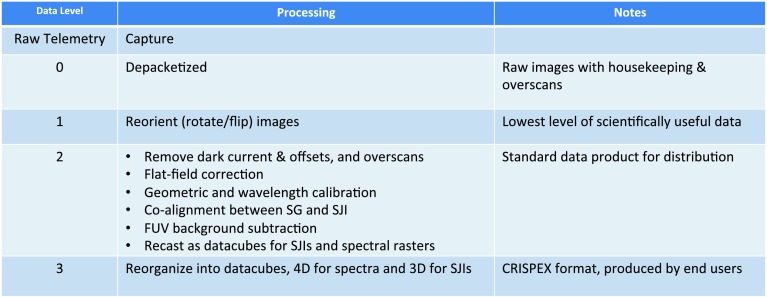
Flow chart of various IRIS data levels and associated pipeline processing.

**Figure 33:**
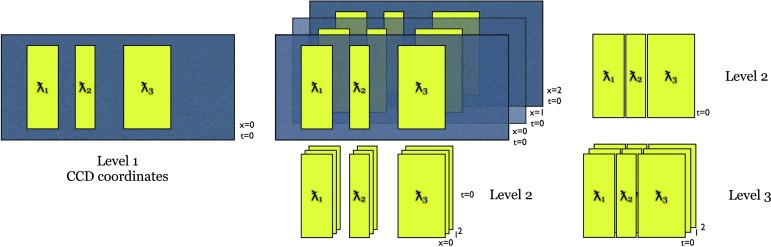
IRIS spectral data layout for various data levels. *Left*: Levels 0 and 1 spectral data have up to eight windows appropriately placed within a pixel array matching the CCD detector. *Middle*: Level 2 data have extracts of the eight windows, assembled into rasters based on slit position $x$. *Right*: Level 3 data assemble the Level 2 rasters into time-series datacubes.

In this section, we describe the flow of IRIS data through the processing pipeline from raw telemetry to data products for distribution to the science community. As summarized in Figure 32, IRIS has four data levels that are processed sequentially as follows:

i) Raw telemetry is captured and converted into Level 0 image files.
ii) Images are rotated and flipped to produce Level 1 data, for which all relevant spacecraft and instrument telemetry is incorporated into the FITS headers. This constitutes the lowest level of scientifically useful data.
iii) The next step is key in the data processing pipeline, where a series of calibration corrections are applied to produce Level 2 data, which is the product released to the public. The type of processing depends on whether the data come from the slit-jaw imager or the spectrograph.
  In general, darks and pedestal offsets and overscan rows are removed, flat-fielding corrections for telescope and CCD properties are applied, and the background in FUV spectral images is subtracted. In addition, geometric and wavelength corrections are applied, so that all images are mapped to a common spatial plate scale and an “ideal” CCD. Spectral images are also remapped to align with an equal-sized array where wavelength and spatial coordinates align with the grid. An array mapping the wavelength axis to physical wavelength is created in this process. We refer to relevant sections of this article for technical details of these calibrations.
  Finally, the data are rearranged and saved as Level 2 FITS files. The rearrangement is based on the spatial and spectral windows defined in the observing sequence (OBS) and depends on the type of data – SJIs or spectrograph rasters: Level 2 SJI-files are time series. That is, all SJIs of the same wavelength channel are put together in one file of a 3D cube by $(x, \, y, \, t)$. Here $x$ and $y$ are spatial dimensions in the direction of raster scan and along the slit, respectively, and $t$ is time. The SJI-images are padded in the $x$ dimension, so that the full field-of-view (FOV) is included. Each single SJI-image is then placed in its appropriate position within this padded area. Each spectral window is saved in its own cube, so that each raster file contains one cube *per* window. The axes of these cubes are $\lambda $ (wavelength), $y$, $x$. Each raster repetition is saved in a separate file. For sit-and-stare observations, there is only one raster repetition, *i.e.* all rasters are in one file.
iv) On the data user end, Level 2 data can be reorganized by SolarSoft tools into Level 3 data for further analysis with the CRISPEX software package (see ITN 26 at http://iris.lmsal.com/itn26/itn26.pdf). Level 3 datacubes are 3D in $(x, \, y, \, t)$ for SJI data and 4D in $(\lambda , y, \, x, \, \,t)$ for spectral data. Figure 33 shows a schematic of the spectral data layouts at various levels.

Processing from Level 1 to 2 is carried out in the data processing pipeline through calls to the IDL routine iris_prep.pro in the SolarSoft IRIS package. iris_level1to2_driver2.pro is the top-level driver and takes two passes for each OBS:

i) In the first pass, it invokes iris_prep with explicit keyword settings to generate a database for various calibrations.
ii) In the second pass, it invokes iris_level1to2.pro, which calls iris_prep with inherit and keyword pipeline=1 to apply the actual corrections using the database from the first pass. iris_level1to2 then rearranges the data and saves them in Level 2 FITS files.

In terms of timing, fresh telemetry is processed in near real time (NRT) with data semi-calibrated. NRT data are a transitory, quicklook product, not released to the public, and are used to produce images and movies posted on the “IRIS Recent Observations” webpage. The full pipeline processing takes place within a few days to produce the final Level 2 data, and the online images and movies are then updated accordingly. When applying various time-dependent corrections, we use the results of the calibration runs that were carried out closest in time to the observations.

IRIS data are occasionally reprocessed when problems with data quality are discovered or calibrations are improved. Data users are advised to download the latest data for their science analysis. We encourage the general science community to report IRIS data issues to iris_calib@lmsal.com. Note that, however, iris_prep is not intended to be used by individual end users because of the complexity involved in the Level 1 to 2 processing and various housekeeping data and because the huge, intermediate database is not being distributed.

## Idiosyncrasies and Known Problems

Despite careful calibrations and data processing, there are still some minor problems in the final IRIS data product. Some of these problems have been corrected in the recent mission-long data reprocessing completed in August 2017, although some still persist and may not be solved soon. While the underlying causes vary from telemetry data dropouts to pipeline software bugs, the majority of these problems, especially those that are ongoing, have negligible to minor impact on science data in general. We describe below a few well-understood problems. A complete list of IRIS idiosyncrasies, together with examples and figures, is documented at http://iris.lmsal.com/documents.html.

i) For a limited number of historic datasets, there were artificial jumps or steps in time in the spectral intensity. This occurred when there were missing housekeeping temperature data due to telemetry dropouts, which caused the temperature-dependent dark correction to fail. This issue has been corrected as of March 2017 by interpolating the temperature data from neighboring orbits.
ii) There are vertical stripes in spectroheliograms at a period equal to the number of SJI channels used times the cadence. The peak-to-peak amplitude is only at a negligible ≈ 0.2 DN level. It is usually noticeable in space–time plots of low-intensity continuum spectra, made from observing sequences with alternating FUV and NUV SJI images. This is a result of imperfect correction of the FUV spectrograph background, which depends on the filter-wheel position (see Section 3.1) and creates an intensity dip at the times of the 2796 SJI images. To remove this artifact, one can adopt empirical tools such as those under the SolarSoft package “mosic” at https://sohowww.nascom.nasa.gov/solarsoft/packages/mosic.
iii) There is an upside-down L-shaped feature in FUV-L spectra near the Si IV 1394 line (as shown in Figure 3), which results from the residual of imperfect background subtraction.
iv) There are features of regular geometric shape in off-limb SJI images that move together with the slit during rasters. The intensity of such features is low, usually no more than 2 – 4 DNs, and thus they only show up against a faint, off-limb background. They are due to scattered light in the SJI optical paths and are very difficult to remove. Examples of such features include i) a ghost of the solar limb (arc) on the left plus a vertical step on the right, appearing off the eastern limb; ii) a bright donut shape with a dark, central vertical bar running across it, appearing off the western limb; and iii) a circular arc of the North Pole.
v) The SG detector burn-in at the C ii lines is currently not being corrected for (see Section 4.3).

## Summary and Conclusions

We have provided a detailed description of various important calibrations applied to IRIS data, including dark correction, scattered light and background correction, flat fielding, geometric distortion correction, wavelength calibration, throughput trending, and wobble corrections. Many aspects of the calibrations have improved substantially since launch, and recent reprocessing of the IRIS Level 2 data archive has made these improvements available to early IRIS data as well. Using the latest version of Level 2 data is therefore important, and SolarSoft routines reading IRIS Level 2 data now alert the user if an outdated version of a data file is being ingested. There remain a few minor calibration issues and idiosyncrasies, as described in this article.

The IRIS team has been and will continue to constantly monitor the data quality and make improvements to the calibration procedures and data processing pipeline on a regular basis to accommodate the evolving instrument characteristics over the mission and to meet the ever-growing needs of the science community. New users should consult the Guide to IRIS Data Analysis, IRIS Technical Note (ITN) 26. It is found online under http://iris.lmsal.com/documents.html, together with other documents of interest. This article supersedes most of the calibration ITNs found there, but relevant ones will be updated as calibrations evolve.
